# A splenic IgM memory subset with antibacterial specificities is sustained from persistent mucosal responses

**DOI:** 10.1084/jem.20180977

**Published:** 2018-08-06

**Authors:** Simon Le Gallou, Zhicheng Zhou, Lan-Huong Thai, Remi Fritzen, Alba Verge de los Aires, Jérôme Mégret, Philipp Yu, Daisuke Kitamura, Emmanuelle Bille, Fabiola Tros, Xavier Nassif, Alain Charbit, Sandra Weller, Jean-Claude Weill, Claude-Agnès Reynaud

**Affiliations:** 1 Team “Development of the Immune System,” Institut Necker-Enfants Malades, Institut National de la Santé et de la Recherche Médicale U1151-Centre National de la Recherche Scientifique UMR 8253, Faculté de Médecine Paris Descartes, Université Paris Descartes, Sorbonne Paris Cité, Paris, France; 2 Flow Cytometry Core Facility, Structure Fédérative de Recherche Necker, Institut National de la Santé et de la Recherche Médicale US24-Centre National de la Recherche Scientifique UMS 3633, Faculté de Médecine Paris Descartes, Université Paris Descartes, Sorbonne Paris Cité, Paris, France; 3 Institute of Immunology, Philipps-Universität Marburg, Marburg, Germany; 4 Division of Molecular Biology, Research Institute for Biomedical Sciences, Tokyo University of Science, Noda, Chiba, Japan; 5 Team “Pathogeny of Systemic Infections”, Institut Necker-Enfants Malades, Institut National de la Santé et de la Recherche Médicale U1151-Centre National de la Recherche Scientifique UMR 8253, Faculté de Médecine Paris Descartes, Université Paris Descartes, Sorbonne Paris Cité, Paris, France; 6 Service de Microbiologie, Hôpital Necker-Enfants Malades, Assistance Publique-Hôpitaux de Paris, Paris, France

## Abstract

To what extent immune responses against the gut flora are compartmentalized within mucosal tissues in homeostatic conditions remains a much-debated issue. We describe here, based on an inducible AID fate-mapping mouse model, that systemic memory B cell subsets, including mainly IgM^+^ B cells in spleen, together with IgA^+^ plasma cells in spleen and bone marrow, are generated in mice in the absence of deliberate immunization. While the IgA component appears dependent on the gut flora, IgM memory B cells are still generated in germ-free mice, albeit to a reduced extent. Clonal relationships and renewal kinetics after anti-CD20 treatment reveal that this long-lasting splenic population is mainly sustained by output of B cell clones persisting in mucosal germinal centers. IgM-secreting hybridomas established from splenic IgM memory B cells showed reactivity against various bacterial isolates and endogenous retroviruses. Ongoing activation of B cells in gut-associated lymphoid tissues thus generates a diversified systemic compartment showing long-lasting clonal persistence and protective capacity against systemic bacterial infections.

## Abstract (precis)

Le Gallou et al. use an AID fate-mapping model to identify an IgM memory population in the spleen of unimmunized mice, originating from persistent gut immune responses and endowed with cross-reactivity against bacteria.

## Graphical Abstract

**Figure:**
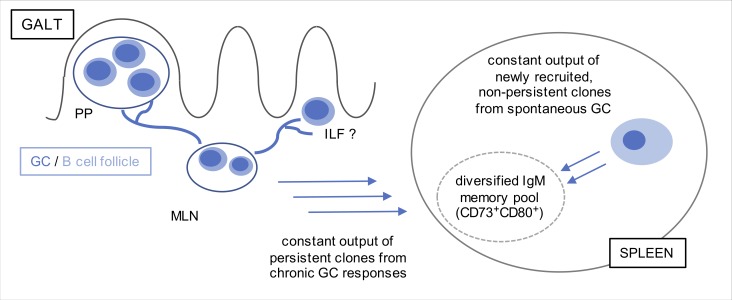

## Introduction

Gut microbiota triggers activation of multiple myeloid and lymphoid effector cells, which in turn prevent their systemic dissemination. Symbiosis is achieved through local cues that contribute to the compartmentalization of mucosal immune responses and to the systemic ignorance of gut commensals in homeostatic conditions (Belkaid and Hand, 2014).

Such a compartmentalization of immune responses occurs at first through the limited translocation of bacteria, sampled from the gut lumen either through dendritic cells carrying them to the mesenteric lymph node (MLN), or through M cell–mediated transcytosis in Peyer’s patches (PPs). The notion of “mesenteric firewall,” proposed by MacPherson et al., refers to such containment of the gut flora, restricting their dissemination and preventing a global activation of the systemic immune system outside inflammatory conditions (Hooper and Macpherson, 2010). Nevertheless, multiple pieces of evidence have been brought recently indicating that bacterial products find their way to peripheral lymphoid organs and profoundly impinge on systemic immune activation. For what concerns B cells, short chain fatty acids, bacterial metabolites, or products of mucosal immune reactions has been described as global or antigen-specific modulators of IgA, IgM, or even IgG antibodies present in the general circulation (Proietti et al., 2014; Gomez de Agüero et al., 2016; Kim et al., 2016; Zeng et al., 2016).

Chronic activation of mucosal B cells takes place in PPs or isolated lymphoid follicles to fuel an IgA-secreting plasma cell compartment in the lamina propria. Such IgAs secreted in the gut lumen exert a potent barrier effect and, through their specific antigen recognition, can target distinct bacterial species, identified through their differential IgA coating (Palm et al., 2014; Bunker et al., 2015). The dependence of B cells from the systemic compartment, notably IgA plasma cells, on mucosal reactions has only recently started to be assessed. Circulating IgAs are reduced in germ-free mice, but such a reduction has been essentially attributed to the massive reduction in the IgA-secreting plasma cell pool observed in the lamina propria (Lécuyer et al., 2014). IgA plasma cells emigrating from the gut have been identified in breast tissues during lactation, an occurrence that corresponds to a specific activation stage (Lindner et al., 2015), and antigen-specific IgA plasma cells have also been detected in bone marrow (BM) after mucosal immunization (Bemark et al., 2016; Lemke et al., 2016). In humans, in whom obviously inflammatory episodes cannot be excluded even in healthy subjects, IgA plasma cells with mucosal markers have been described in BM, and a residual IgA plasmablast population with similar markers has been observed in the blood upon rituximab treatment, suggesting ongoing output from rituximab-resistant mucosal plasma cell progenitors (Mei et al., 2009, 2010).

The group of D. Allman recently reported the presence of BM IgA plasma cells harboring antibacterial specificity in the absence of external stimuli, a subset whose formation required the gut flora (Wilmore et al., 2018). Clonal relationships were also described between gut IgA plasma cells and spleen memory B cells (Lindner et al., 2015), indicating that such mucosal–peripheral crosstalk can take place in a homeostatic context. To more globally assess relationships of peripheral B cells to mucosal immune reactionsoutside inflammatory conditions or immunization, we used lineage tracing of AID-experienced cells, by marking B cells engaged in immune responses in a time-controlled manner (Dogan et al., 2009). We report here that in healthy, nonimmunized mice raised in a clean animal facility, a long-lasting splenic IgM (and smaller IgA) compartment harboring mutated Ig genes and specificities against antigens from bacteria and endogenous retroviruses (ERVs) is maintained through the constant input of B cell clones persisting in PP germinal centers (GCs) and constitutes a pool of preactivated B cells that can be rapidly mobilized upon infectious challenges.

## Results

### A persistent AID-labeled B cell population in nonimmunized mice

**Figure 1.**
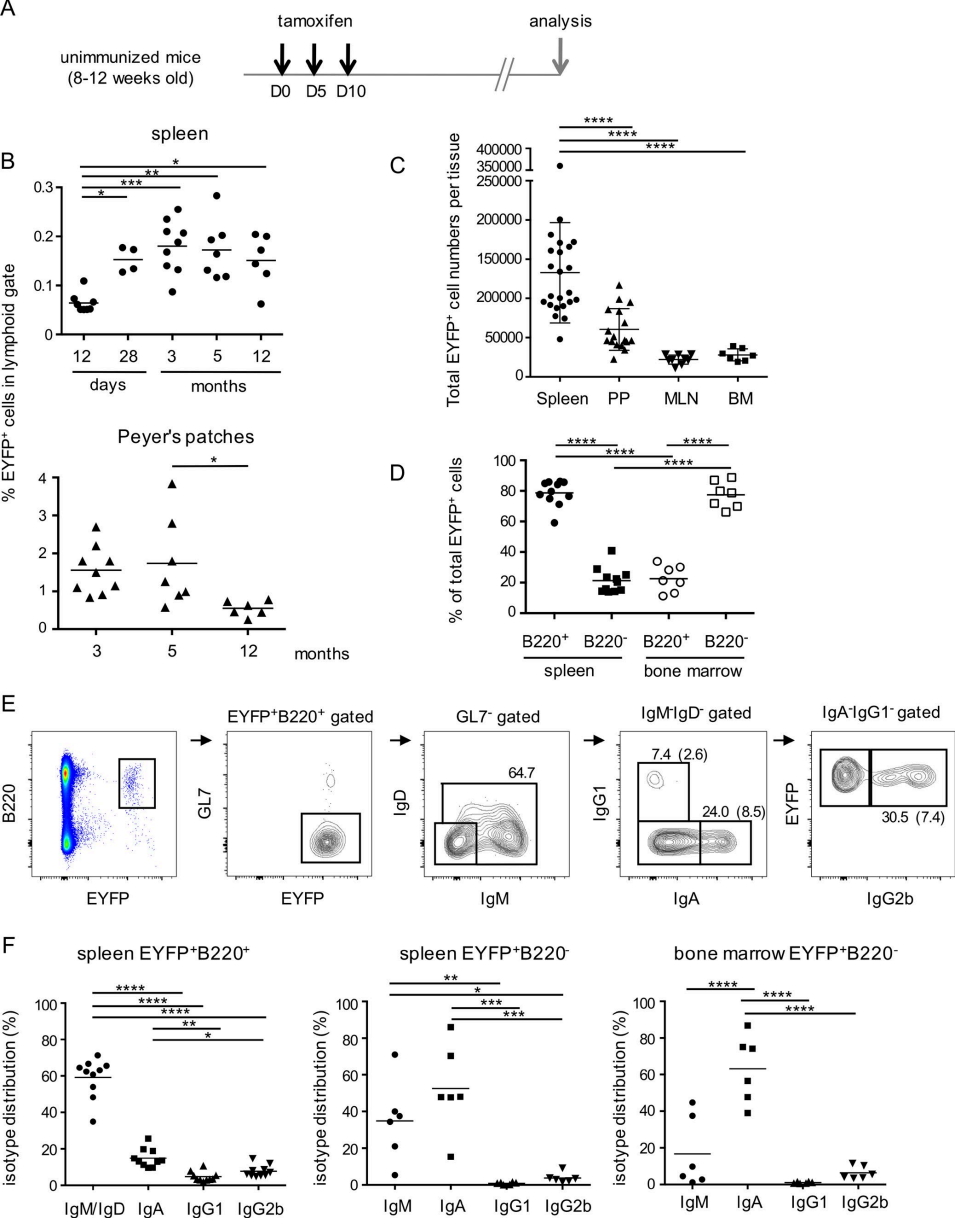
**A spontaneous, persistent immune response in absence of deliberate immunization is observed in the AID-Cre-ERT2xROSA26-loxp-EYFP (“AID-Cre-EYFP”) reporter mouse upon tamoxifen ingestion. (A)** A three tamoxifen feeding schedule, 5 d apart, was used to identify spontaneous AID-expressing cells in AID-Cre-EYFP mice. **(B)** Evolution of EYFP^+^ cell fraction with time in spleen and PPs. Time in months is ± 10 d, and in days refer to the first tamoxifen gavage (D0). **(C)** Total EYFP^+^ cell numbers per organ, 3 mo after the last tamoxifen ingestion, in spleen, PPs, MLNs, and BM (BM cell counts represent two femurs per animals and are thus a partial estimate of the total cell population). **(D)** Distribution of EYFP^+^ cells among B220^+^ and B220^−^ subsets in spleen and BM, 3–4 mo after tamoxifen feeding. **(E)** Representative FACS profiles of heavy chain isotype distribution among spleen EYFP^+^ cells. Percentages are indicated for each panel, with values in brackets referring to the initial B220^+^EYFP^+^GL7^−^ population. **(F)** IgM, IgA, IgG1, and IgG2b isotype distribution among spleen EYFP^+^ memory (B220^+^) and plasma cells (B220^-^) and BM B220^−^ plasma cells, 3–4 mo after tamoxifen gavage. Intracellular staining was performed for plasma cells. *, P < 0.05; **, P < 0.01; ***, P < 0.001; ****, P < 0.0001, one-way ANOVA with Holm-Sidak correction for multiple comparisons. Nonsignificant differences are not indicated. Mean values are indicated, with data from each individual mouse represented. Each analysis originates from two or more independent tamoxifen-labeling experiments. See also Fig. S1.

The AID-Cre-ERT2xROSA26-loxP-EYFP mouse (hereafter named AID-Cre-EYFP) allows the labeling of AID-expressing B cells upon tamoxifen feeding (Dogan et al., 2009). To evaluate the possible contribution of spontaneous/chronic immune reactions to the memory B cell pool, we used an experimental scheme of three tamoxifen ingestions, corresponding approximately to a 9–15-d labeling period (Fig. 1 A; Jarjour et al., 2014). A distinct B cell population was labeled over this time period and persisted several months after its initial formation, with little decay observed until 1 yr in spleen and a twofold decay after 1 yr in PPs (Fig. 1 B). The frequency of EYFP^+^ labeled cells was highest in PPs (1–2% after 3 mo compared with ∼0.2% in spleen), but total cell numbers were more important in spleen (133,000 ± 13,000 cells) than in PPs (60,000 ± 6,400), mesenteric lymph nodes (MLNs, 22,000 ± 2,000), or BM (BM, 28,000 ± 2,900; Fig. 1 C). Very low numbers of EYFP^+^ cells were observed in other lymph nodes (inguinal or axillary), as well as in the circulation (Fig. S1 A). These numbers should be corrected for the labeling efficiency, as tamoxifen only induces Cre-mediated EYFP^+^ expression in 20–50% of AID-expressing cells (Dogan et al., 2009; Tas et al., 2016). The persistent EYFP^+^ population comprised memory B cells and plasma cells in the spleen in an ∼4:1 ratio and in a reverse proportion in BM (Fig. 1 D). Memory B cells were mainly IgM^+^ in spleen, with a smaller IgA fraction (Fig. 1 E). Other isotypes included IgG2b and IgG1, while very few cells expressed IgG3 and almost none expressed IgG2c (not shown). For plasma cells, IgA^+^ cells were more abundant and clearly dominated over other isotypes in BM (Fig. 1 F).

Insertion of the Cre recombinase disrupted the *Aicda* locus, making the reporter line haploinsufficient for AID. We therefore designed as control a reporter model in which the same targeting construct was used to modify a bacterial artificial chromosome (BAC) including 190 kb around the *Aicda* gene and obtained two transgenic lines with one copy each. EYFP-labeled B cell numbers were slightly, but not significantly, lower compared with the knock-in mouse, 3–5 mo after labeling, indicating that *Aicda* haploinsufficiency has only a moderate impact on this AID-dependent spontaneous response (Fig. S1 B). The knock-in AID-Cre-EYFP mouse line, devoid of extra copies of linked genes present in the BAC construct, was therefore used throughout this study.

### The endogenous response originates from GC B cells

**Figure 2.**
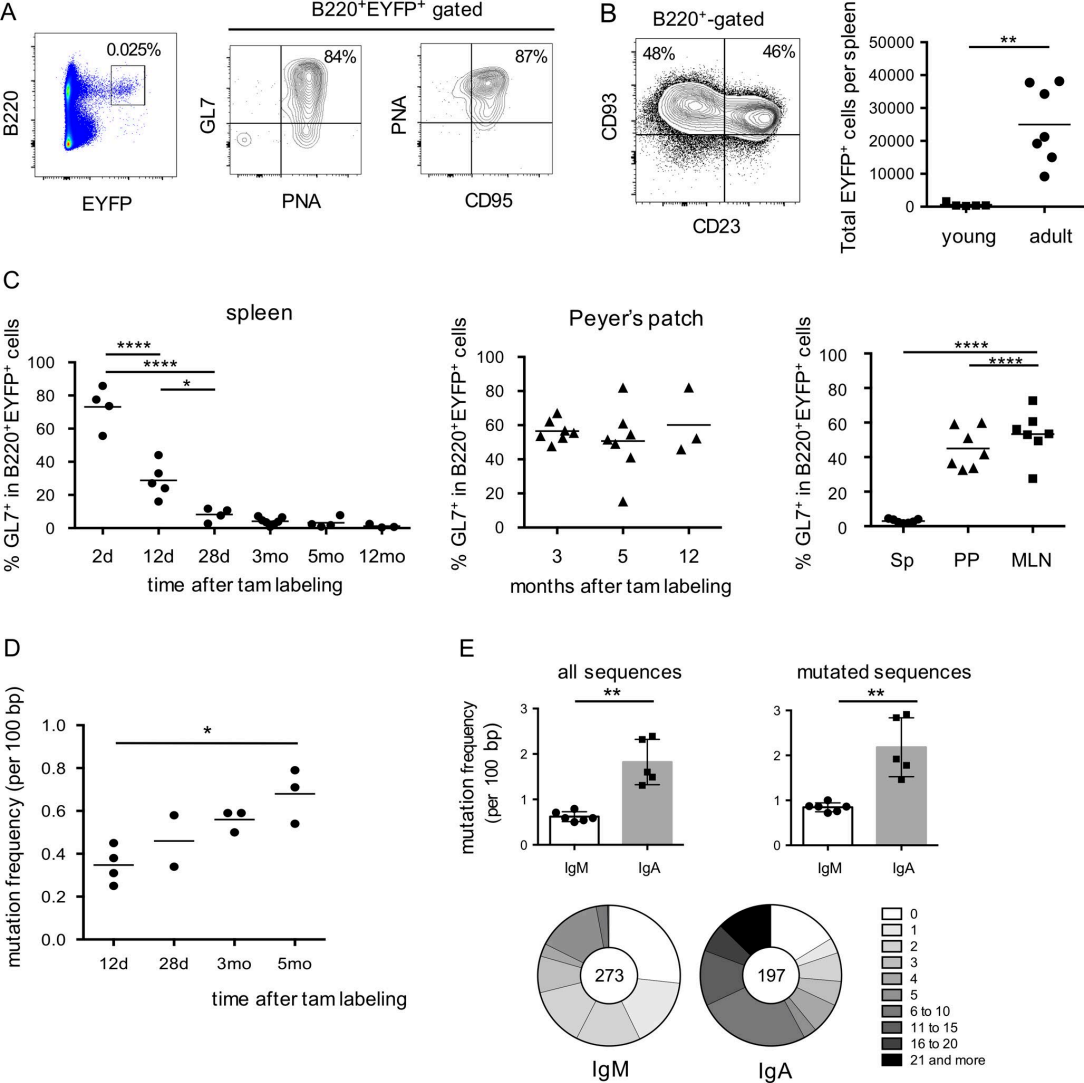
**Kinetics of AID-dependent labeling within GCs show clonal persistence in PPs and MLNs and V_H_ gene mutation accumulation in spleen. (A)** GL7, PNA and CD95 phenotype of splenic B cells, 2 d after tamoxifen feeding. **(B)** Tamoxifen feeding of 20-d-old mice does not mark transitional B cells. Left, representative FACS profile of splenic B220^+^ cells from young mice (20-d-old), displaying a T1 (CD93^+^CD23^−^) and T2 (CD93^+^CD23^+^) phenotype. Right, total number of splenic EYFP^+^ cells, 2 d after a single tamoxifen ingestion in young (20 d old) versus adult mice. See also Fig. S2 (C). Fraction of EYFP^+^ with a GC phenotype as function of time after tamoxifen labeling. Left, spleen (day 0 corresponds to the first tamoxifen feeding, the 2d point thus corresponding to a single tamoxifen ingestion); middle, PP; right panel, comparison of different tissues, 3–4 mo after tamoxifen feeding. For the spleen panel, significant differences are only indicated for the first three time points. **(D)** Mutation frequency among splenic EYFP^+^ IgM^+^ B cells (rearranged intronic J_H_4 sequences), from mice analyzed at various time points after tamoxifen labeling. **(E)** Mutation frequency and distribution among splenic EYFP^+^ IgM^+^ and IgA^+^ subsets, from mice analyzed between 3 and 5 mo after tamoxifen feeding. The total number of sequences is indicated in the pie chart, with a distribution of sequences with a given number of mutations. *, P < 0.05; **, P < 0.01; ****, P < 0.0001, Student's *t* test for panels B and E or one-way ANOVA with Holm-Sidak correction for panels with multiple comparisons. Mean values are indicated. All mice analyzed are represented and correspond to two or more independent experiments.

Mouse transitional B cells have been described as expressing low but distinct levels of AID (Kuraoka et al., 2011), and we therefore studied at which stage of B cell development the Cre-ERT2 enzyme was activated. To this end, we analyzed splenic B cells 48 h after a single tamoxifen feeding in 2–3-mo-old animals. >75% of spleen EYFP^+^ B cells were GL7^+^, and almost all were PNA^+^ or CD95^+^ (Fig. 2, A and C). Localization of PNA^+^EYFP^+^ in GC structures was observed in both spleen and PPs (Fig. S2). We performed the same short-term labeling for 20-d-old animals, at which stage the vast majority of spleen B cells is composed of T1 and T2 immature B cells (Fig. 2 B). In these conditions, the EYFP labeling was minimal, clearly indicating that AID expression level at the transitional stage is insufficient to allow for Cre recombinase activity at the reporter locus (Fig. 2 B). EYFP labeling thus marks cells engaged in spontaneous/chronic GCs.

The follow up of GL7 labeling with time showed a decay of GL7^+^EYFP^+^ cells within a few days, indicating maturation of labeled cells in the spleen (Fig. 2 C, left). In contrast, analysis of PPs revealed a persistent EYFP^+^GL7^+^ population over one year, i.e., a long-lasting persistence of clones induced by chronic responses within GCs (Fig. 2 C, middle). Persistence of EYFP^+^ cells with a GC phenotype was also observed in MLNs (Fig. 2 C, right). Interestingly, whereas GL7^+^ labeling dropped down rapidly in the spleen, the splenic EYFP^+^ memory population increased from day 12 to 3 mo after labeling, at which time it reached a plateau (Fig. 1 B), suggesting that import from other sites of EYFP^+^ B cell formation may contribute to the accumulation of the splenic memory pool.

Mutations estimated on J_H_4 intronic sequences indicated a clear, but moderate mutation load in the IgM pool (0.62%), with 27% of unmutated sequences in this AID-labeled subset and threefold higher mutation values for spleen IgA^+^ B cells (1.82%) in animals studied 3–5 mo after tamoxifen feeding (Fig. 2 E). Mutation frequency increased from day 12 to 5 mo after labeling in the IgM^+^EYFP^+^ population (Fig. 2 D). The EYFP^+^B220^+^ population represents thus antigen-experienced B cells, marked and diversified by AID during activation in GCs.

### A large IgM memory B cell pool accumulates with age and requires T cell– and TLR-dependent signals

**Figure 3.**
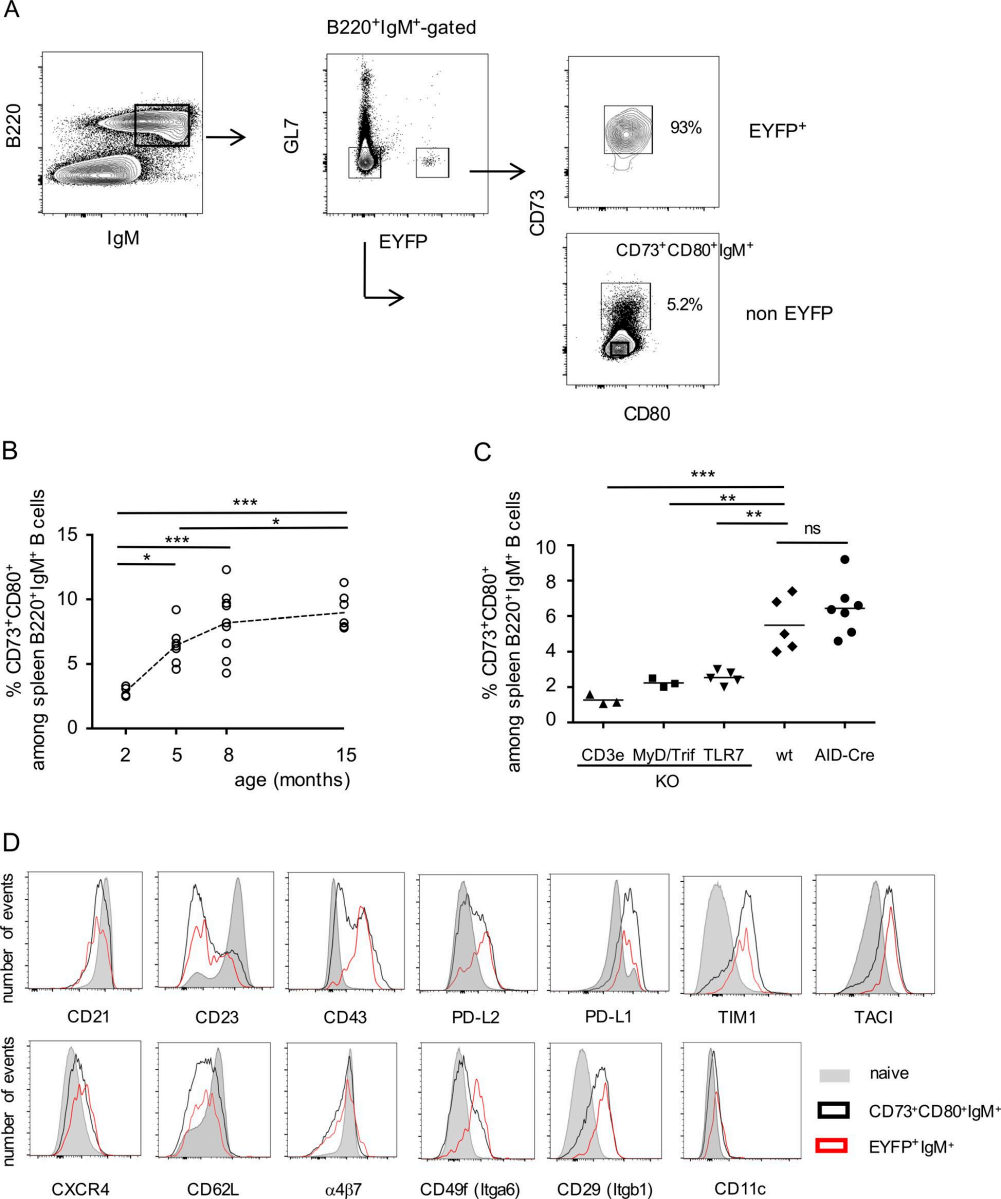
**A large mutated IgM B cell pool with memory marker expression accumulates with age in the spleen and shows T cell and TLR dependence. (A)** Gating strategy used to defined the total IgM^+^CD73^+^CD80^+^ subset: a CD73^+^CD80^+^ gate including most EYFP^+^ cells is set on the B220^+^IgM^+^GL7^−^EYFP^+^ population (upper right), and then used to define the percentage of non-EYFP^+^ cells in this gate (lower right). **(B)** Evolution of the total IgM^+^CD73^+^CD80^+^ compartment with time (based on a gate set as defined in A). Ig gene mutations (rearranged intronic J_H_4 sequences) are shown in Table S1. **(C)** Frequency of IgM^+^CD73^+^CD80^+^ B cells in T cell and TLR-deficient backgrounds, compared with age-matched, 4-mo-old, wild-type, and AID-Cre-EYFP mice (with a preset gate on EYFP^+^B220^+^GL7^−^IgM^+^ cells from a parallel analysis). *, P < 0.05; **, P < 0.01, one-way ANOVA with Holm-Sidak correction. Mean values are indicated. Cumulative data of all analyzed animals are indicated. **(D)** Representative FACS profiles of naive (B220^+^IgM^+^EYFP^−^GL7^−^CD73^−^CD80^−^), B220^+^GL7^−^CD73^+^CD80^+^ EYFP^+^ and EYFP^−^ subsets (gates represented in A [right], including for naive B cells).

Interestingly, all IgM^+^EYFP^+^ B cells harbored a double CD73^+^CD80^+^ phenotype, which has been described as the hallmark of mature memory B cells (Fig. 3 A; Taylor et al., 2012; Zuccarino-Catania et al., 2014). We took advantage of this phenotype to evaluate the size of the total, non-EYFP, IgM “memory” population in the spleen. By setting a gate on the EYFP^+^ population in a CD73/CD80 FACS analysis, we could estimate the proportion of non-EYFP B cells in the same gate (Fig. 3 A). This CD73^+^CD80^+^IgM^+^ population was then followed according to mouse age, and appeared to increase markedly up to 5 mo of age (6% of total B220^+^ B cells), while reaching progressively a plateau at later time points (7.9 and 8.7% at 8 and 15 mo, respectively; Fig. 3 B). Mutations were analyzed in the total IgM^+^CD73^+^CD80^+^ population sorted with a preset gate on EYFP^+^ cells. The higher proportion of unmutated sequences compared with the EYFP^+^IgM^+^ subset (Table S1) suggests that some naive B cells are included in this gate (∼25%), leading to a corresponding slight overestimation of the CD73^+^CD80^+^ B cell pool. When mutated sequences are considered, the mutation frequency was still slightly lower than the one of mutated sequences from the IgM^+^EYFP^+^ subset (0.66 vs. 0.85%, Table S1 and Fig. 2 D).

Using a preset gate on EYFP^+^ B cells from a reference mouse, we also estimated the frequency of CD73^+^CD80^+^ B cells in CD3ε-, MyD88xTrif- and TLR7-deficient mice (Fig. 3 C), and compared it to wild-type mice. This population was impacted in all cases, most strongly in T cell deficient mice, indicating that both T cell and TLR-derived signals contribute to the formation of this mutated, memory-like subset.

We then analyzed the EYFP^+^ and EYFP^−^ CD73^+^CD80^+^IgM^+^ population for a set of activation markers, chemokine receptors or integrins whose expression differed from naive B cells (Fig. 3 D). Both populations largely lacked CD23 expression, and showed reduced (but positive) CD21 expression levels compared with the naive subset. They showed a similar increased expression of activation markers like TIM-1 or PD-L1 and reduced expression of recirculation markers like CD62L or α4β7 integrins. CD11c was not markedly up-regulated, which, together with CD21, clearly distinguished these subsets from aged B cells (Hao et al., 2011; Rubtsov et al., 2011). EYFP^+^ and EYFP^−^ subsets differed for other memory/activation markers, with higher expression of CD43, PD-L2 or TACI in EYFP^+^ B cells, as well as two integrins, α6 and β1, reported to mediate binding to the extracellular matrix in lymphoid tissues (Song et al., 2013). Altogether, the EYFP^+^ and EYFP^−^ CD73 CD80 compartments share enhanced memory/activation marker expression compared with naive B cells, together with reduced hallmarks of recirculation and enhanced ones for residency, some of these features being more pronounced in EYFP^+^ B cells.

### The endogenous response in spleen originates from B2 cells

**Figure 4.**
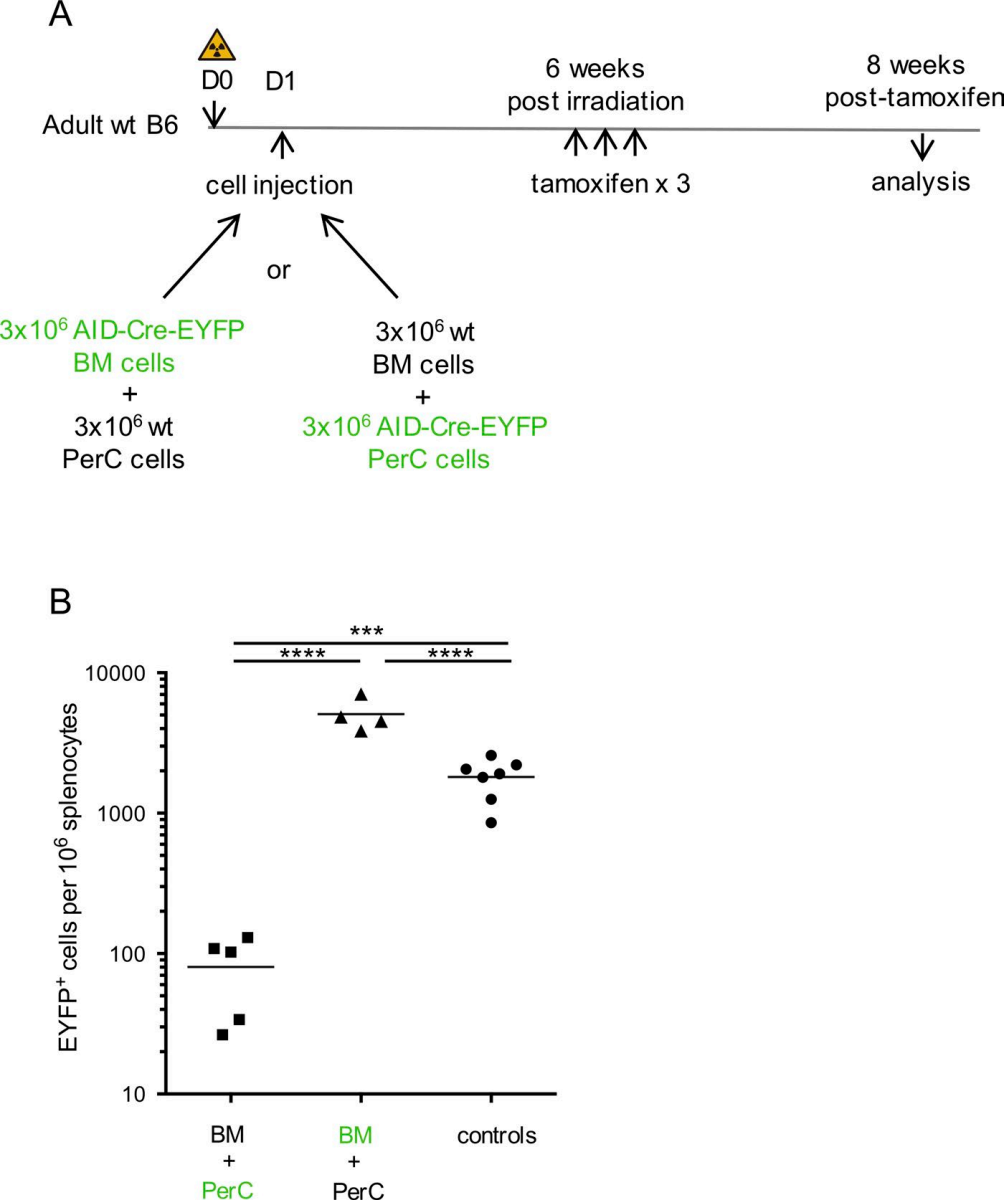
**The spontaneous immune response originates from B2, not B1 cells. (A)** Scheme of restoration with cells from BM and peritoneal cavity from either wild-type or AID-Cre-EYFP origin after sublethal irradiation of the host and time frame of experiment. **(B)** Number of splenic EYFP^+^ cells per 10^6^ lymphoid cells observed in restored animals, with tamoxifen-fed AID-Cre-EYFP animals as controls (according to Fig. 1 A protocol). Data represent two separate experiments; analysis performed at other time points gave similar results. See also Fig. S3. ***, P < 0.001; ****, P < 0.0001.

B1 B cells are considered to be the main providers of natural antibodies, and, more globally, to be spontaneously activated cells. Moreover, some markers observed on IgM^+^EYFP^+^ B cells, like CD43, are often used to identify B1 B cells in the spleen, and TIM-1^+^ cells are enriched among CD5^+^ and regulatory B cells (Xiao et al., 2012). To ask whether the endogenous response observed is of B1 or B2 origin, we restored sublethally irradiated mice with i.v. injection of mixtures of cells from BM and peritoneal cavity, coming from wild-type or AID-Cre-EYFP mice in either of the two combinations (Fig. 4 A). Tamoxifen was given 6 wk after cell transfer and analysis performed 8 wk after tamoxifen labeling.

A large memory B cell subset was labeled when BM cells from AID-Cre-EYFP mice were used in the transfer, while minimal labeling was observed when the reporter line was used as source of peritoneal cavity B cells (Fig. 4 B). Interestingly, a distinct labeling of B1b cells in the peritoneal cavity was observed upon restoration with AID-Cre-EYFP BM cells and also in control AID-Cre-EYFP fed with tamoxifen, indicating that a fraction of B1b cells can express AID (Fig. S3). The endogenous response thus appears to have mainly a B2 origin.

### IgM diversification and switching to IgA requires the gut flora

The spontaneous appearance of EYFP^+^ B cells in the spleen compartment raises the question of their origin, and the mucosal lymphoid tissue, with its persistent GC clones appears as a possible candidate. We therefore studied the presence of EYFP^+^ in germ-free mice. Tamoxifen feeding proved to be not practicable, with systematic bacterial colonization of the gut observed after a few weeks. We therefore resorted to sterile, irradiated tamoxifen-containing food. A 2-wk feeding protocol was chosen as giving reasonable labeling efficiencies, albeit lower compared with three tamoxifen gavages. However, a large variability was observed between mice, possibly linked to the unpleasant taste of the food and its intermittent consumption. Conventional housed animals fed with the same diet were used as controls.

**Figure 5.**
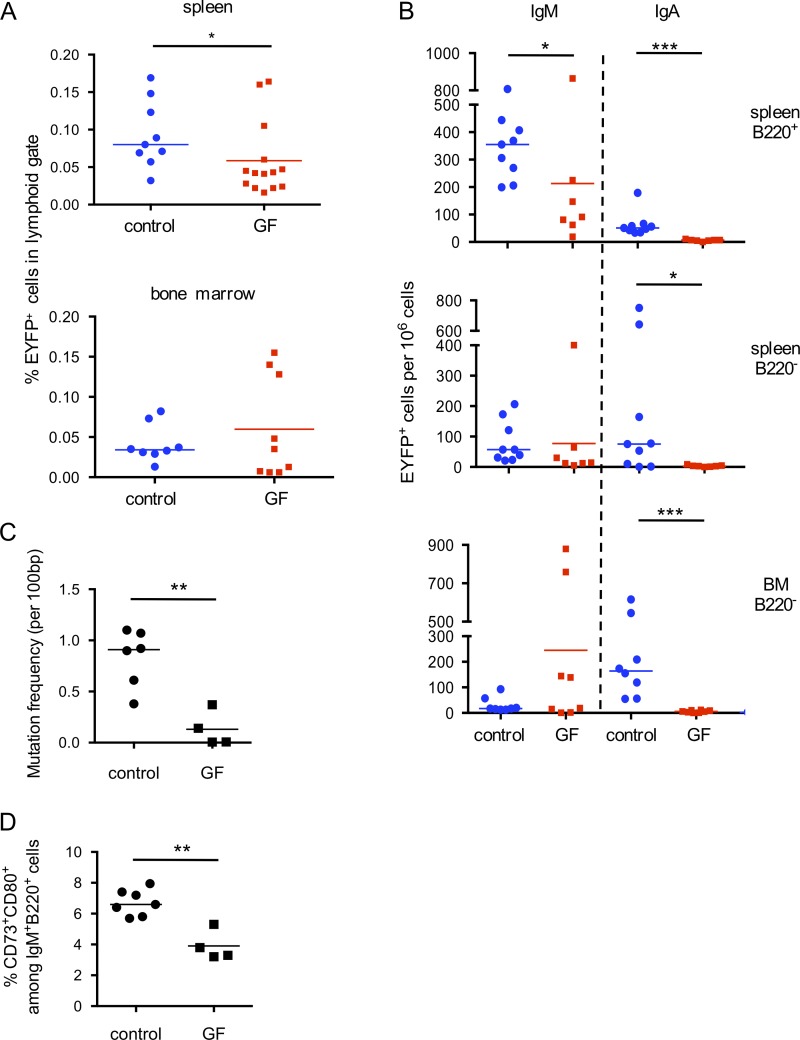
**Memory IgM B cell diversification and switching to IgA requires the gut flora.** Germ-free (GF) and control mice were given tamoxifen-containing food for 2 wk and analyzed 2 mo after the treatment. **(A)** Analysis of total EYFP^+^ cells in spleen (top) and BM (bottom). **(B)** IgM and IgA isotype distribution among spleen EYFP^+^ B cells (B220^+^) and spleen and BM plasma cells (B220^−^). **(C)** Mutation frequency in heavy chain J_H_4 rearranged sequences from B220^+^IgM^+^EYFP^+^ B cells from germ-free mice and control animals fed the same tamoxifen diet. **(D)** Percentage of CD73^+^CD80^+^ among total B220^+^IgM^+^ splenic B cells from germ-free mice and control animals fed the same tamoxifen diet. *, P < 0.05; **, P < 0.01; ***, P < 0.001, Mann-Whitney test. Mean values are indicated. Animals from two different cohorts were analyzed.

Total EYFP^+^ cell numbers were moderately, but significantly reduced in the spleen of germ-free mice, but the most drastic impact was on the IgA-positive subsets, both IgA memory and IgA plasma cells in spleen and BM (Fig. 5, A and B). For IgM subsets, a significant reduction in cell numbers was observed, and, most strikingly, in the mutation frequency of the IgH locus (Fig. 5 C), indicating that, whereas spontaneous AID activation still occurs in germ-free mice, the GC reaction that gives rise to EYFP^+^ B cells might be more transient. This differential mutation load was confirmed by V_H_ sequencing of hybridomas generated from EYFP^+^IgM^+^ cells of conventional and germ-free mice (see below). Reduction of the total splenic pool of CD73^+^CD80^+^IgM^+^ B cells was accordingly observed (3.9 vs. 6.6%; Fig. 5 D). Thus, whereas splenic EYFP^+^IgM^+^ B cells are reduced but still generated in the absence of the gut flora, the low level of somatic diversification observed suggests that their antigen specificity may differ (see below).

### Similar clones contribute to the PP, splenic, and BM IgM/IgA memory and plasma cell pool

To delineate the relationships between the different compartments, we sequenced different B cell subsets to uncover clones shared between them.

**Figure 6.**
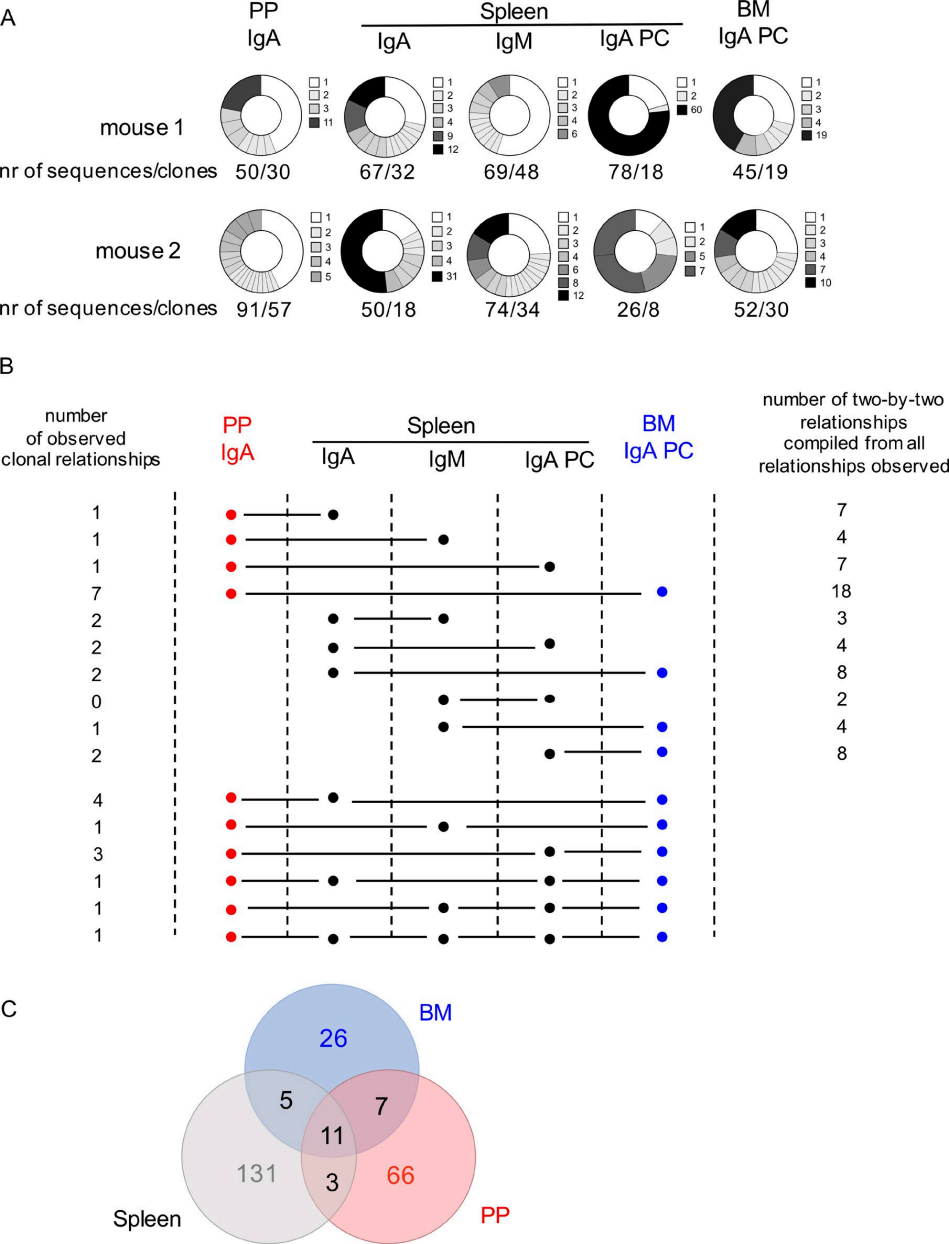
**EYFP^+^ B cell clones are shared between PPs, spleen, and BM, as well as between IgA plasma cells and IgM and IgA memory B cells.** EYFP^+^ PP IgA memory B cells, spleen IgA and IgM memory B cells, spleen and BM IgA^+^ plasma cells (PC) were isolated from two mice, one year after tamoxifen feeding, and rearranged J_H_4 sequences were determined and submitted to CDR3 clustering for clonal relationship analysis between these different subsets. **(A)** Clonal distribution within each subset. Each pie section represents one clone identified through its CDR3 junction, except for the white segment that represents all unique sequences. The total number of sequences and clones is indicated below each pie chart, and the clone size is color-coded for each subset as indicated on the right side. Nr, number. **(B)** Number and tissue/subset/isotype distribution of clones shared between different B cell and plasma cell subsets. The first 10 lines depict clones shared between two subsets, and the last six lines clones present in multiple subsets (not all possible configurations were observed). Numbers on the right side correspond to the sum of two-by-two relationships, including those observed in multiple clonal configurations. **(C)** Venn diagram of clones shared between spleen, PPs, and BM (the three subsets analyzed for spleen are pooled, thus slightly reducing the total number of splenic clones compared with A).

We isolated five different EYFP fractions from two mice, 1 yr after tamoxifen labeling: IgA memory B cells from PPs and IgM and IgA memory B cells, as well as IgA plasma cells from spleen and IgA plasma cells from BM. Approximately 60 sequences were obtained for each subset, i.e., 309 and 313 total sequences from each mouse. These 622 sequences segregated into 249 clones, with several showing considerable clonal expansion (a clone distribution per subset is represented in Fig. 6 A). Interestingly, in spite of the modest number of sequences collected from each subset, ∼12% of the clones observed comprised sequences originating from different tissues and/or harboring different isotypes. The clonal relationships observed are displayed in Fig. 6 B and concern all subsets, in various combinations, including clones present in three, four, or all subsets. Number of clonal relationships observed are listed on the left side of Fig. 6 B, with numbers on the right side corresponding to the total number of two-by-two relationships decomposed from clones comprising multiple subsets. Clonal overlap between the different tissues, irrespective of isotype or cell type, is depicted in Fig. 6 C.

These results strongly support a common origin of EYFP^+^ cells from these different tissues, isotypes, and differentiation stages.

### Constant renewal from persistent clones rather than B cell longevity accounts for the persistence of EYFP^+^ cells in the spleen

The persistence of EYFP^+^ cells in PP GCs, the impact of the gut flora and the clonal relationships observed between PPs, spleen, and BM EYFP^+^ cells suggest that constant output from mucosal tissues, rather than cell longevity, may account for the long-term persistence of EYFP^+^ cells at the periphery. Such an observation was recently reported for lamina propria plasma cells, which were shown to be generated by memory B cell clones displaying longitudinal persistence and ongoing diversification (Lindner et al., 2015).

**Figure 7.**
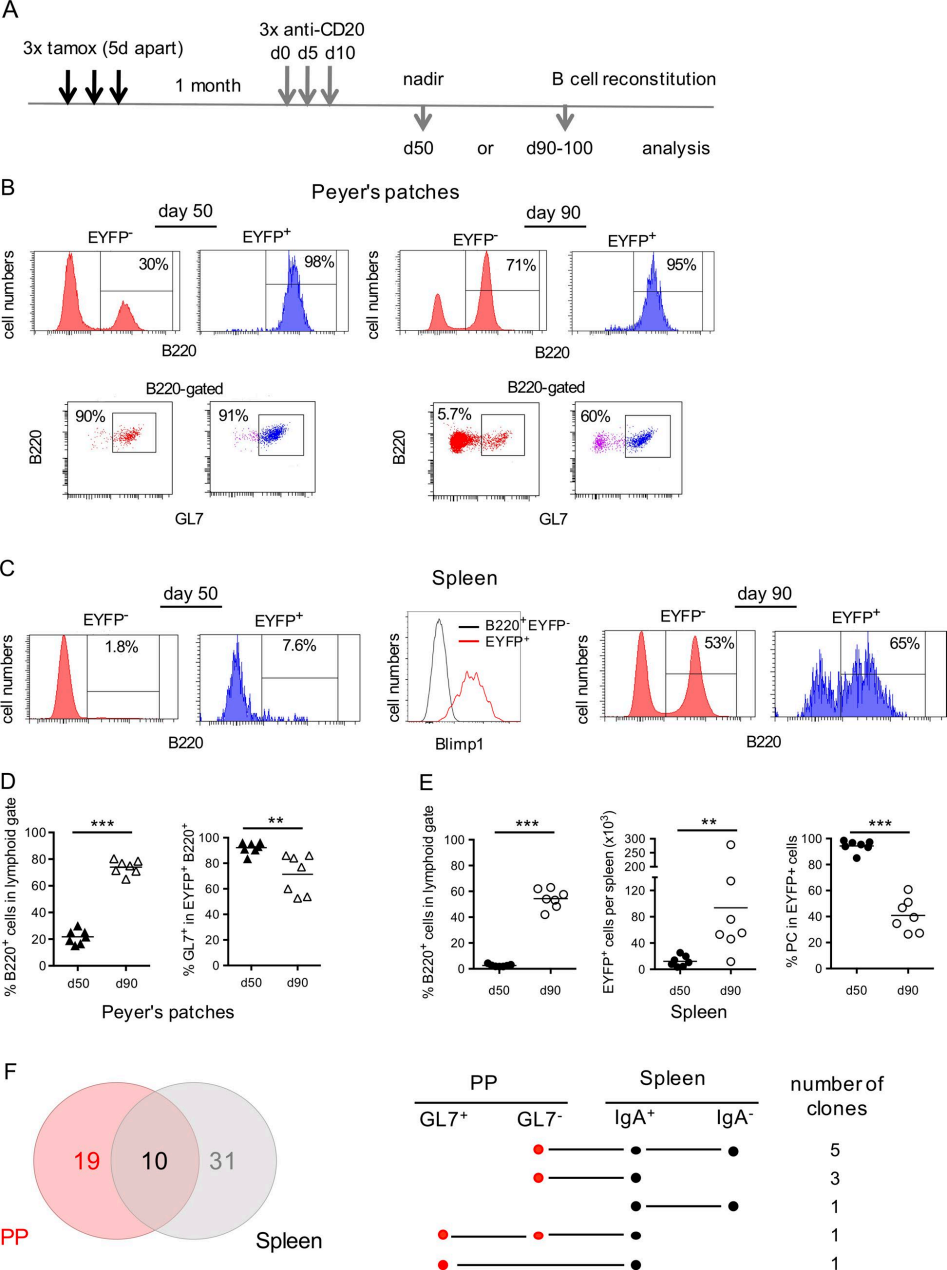
**Anti-CD20 depletion reveals the replenishment of the splenic EYFP^+^ pool from clones persisting within mucosal GC reactions. (A)** Anti-CD20–mediated depletion was performed by three injections, 5 d apart, 1 mo after tamoxifen labeling. **(B and C)** Analysis of PPs (B) and spleen (C) was performed on EYFP^+^ and EYFP^−^ cells at day 50 after the first anti-CD20 injection, which represents the nadir of B cell depletion, and at day 90–107 (day 90), at which time the B cell compartment is largely restored. FACS profiles show that residual EYFP^+^ cells resisting the anti-CD20 treatment are mainly GC B cells in PPs (B, left) and plasma cells in the spleen, with a Blimp-1^+^ profile (C, left and middle), while EYFP^+^ memory B cells reemerge during reconstitution of the B cell pool in both spleen and PPs (B and C, right). **(D)** Percentage of B cells in PPs (left) and frequency of GC B cells among EYFP^+^ cells (right) at both time points. **(E)** Percentage of total (left part) and EYFP^+^ B cells (middle part) in spleen, with the percentage of plasma cells (B220^−^) among EYFP^+^ cells (right panel). **(F)** Clonal relationships observed among GL7^+^ and GL7^−^ EYFP^+^ cells from PPs and IgA^+^ and IgA^−^ EYFP^+^ splenic cells from two individual mice analyzed at day 90 and 107, respectively, after anti-CD20 treatment. Clonal distribution within the different subsets is represented in Fig. S4. The Venn diagram indicates the number of clones shared between both tissues. Mean values are represented. Each symbol represents an individual mouse. **, P < 0.01; ***, P < 0.001, Student's *t* test. Data are from three or more independent experiments.

We used B cell depletion by anti-CD20 antibody to address this issue, taking advantage of the fact that anti-CD20 treatment in the mouse, as opposed to humans, is rather inefficient at depleting GC B cells (Baumjohann et al., 2013; Mahévas et al., 2013). We treated mice by three anti-CD20 injections 5 d apart, 1 mo after tamoxifen feeding (Fig. 7 A). Mice were analyzed either at day 50, which represents the nadir of B cell depletion, or at day 90, at which time the B cell compartment is largely reconstituted. Accordingly, most EYFP^+^ B cells resisting anti-CD20 depletion at day 50 in PPs had a GC phenotype (Fig. 7 B, left; and Fig. 7 D), while EYFP^+^ cells surviving the treatment in the spleen were mainly B220^−^ Blimp-1^+^ plasma cells (Fig. 7 C, left and middle; and Fig. 7 E). Rather strikingly, the restart of lymphopoiesis was accompanied by the reappearance of memory EYFP^+^ cells in spleen, and in PPs as well, consistent with a PP ongoing export upon termination of drug activity (Fig. 7, B and C, right; and Fig. 7, D and E).

To establish a more direct link between EYFP^+^ B cells from PPs and spleen through their clonal relationships, we sorted two different subsets from each tissue, from two different mice having their B cell compartment fully restored (at day 90 and 107 after the first anti-CD20 injection, respectively): GL7^+^ and GL7^−^ B cells from PPs and IgA^+^ and IgA^−^ B cells from spleen. A total of 261 VDJ sequences were obtained, segregating into 41 clones for spleen and 29 clones for PPs (Fig. S4), with 10 clones in common, i.e., ∼1/3 and 1/4 of total clones from PPs and spleen, respectively. Clones shared between tissues involved the different subsets, IgA^+^ or IgA^−^ and GL7^+^ or GL7^−^ (Fig. 7 E). The splenic EYFP^+^ memory compartment is thus replenished after anti-CD20 treatment from mucosal B cells that resisted B cell depletion.

**Figure 8.**
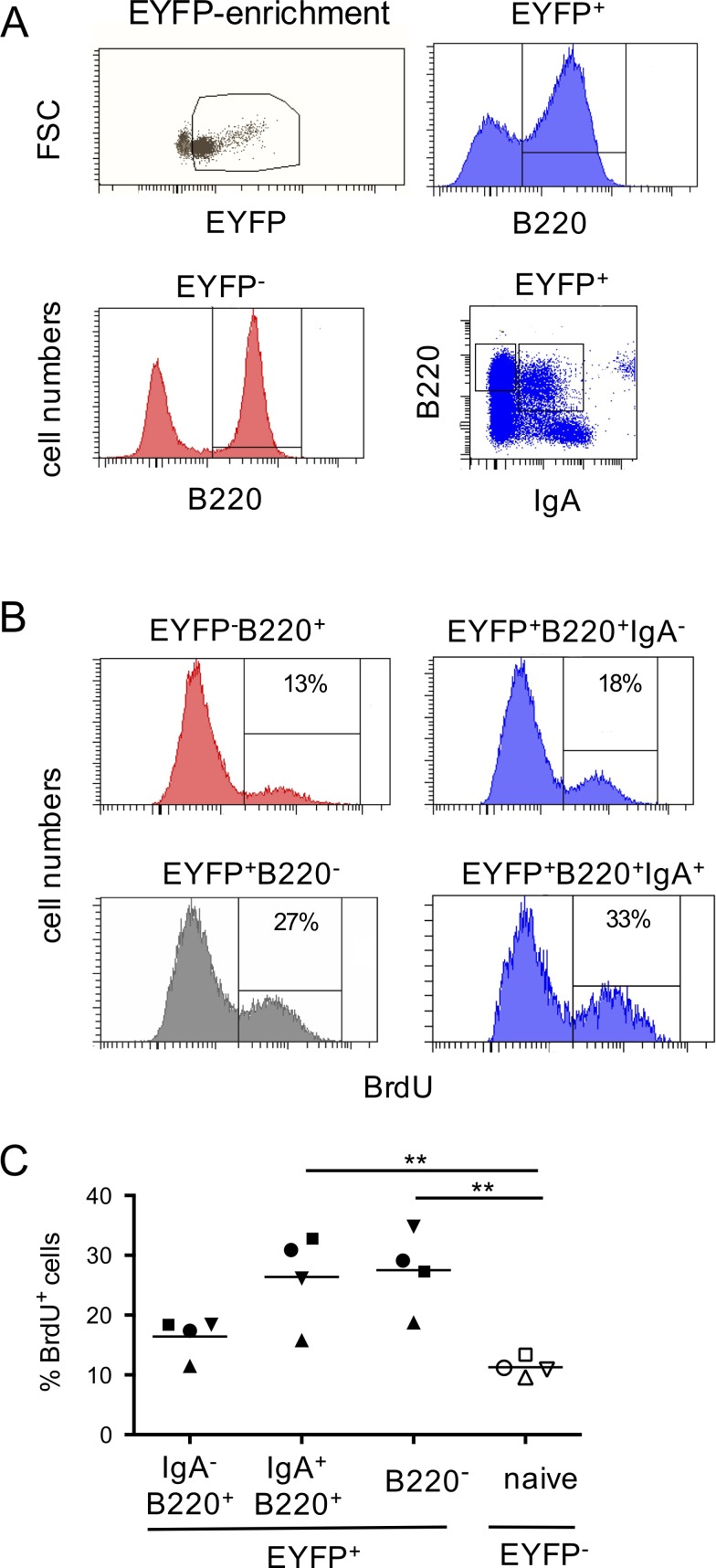
**BrdU labeling shows ongoing replenishment of the EYFP^+^ splenic subset. (A)** An 8-d BrdU pulse, 2 mo after tamoxifen labeling was performed. EYFP^+^ cells were enriched by prior sorting (top panel, left), to allow for a clear distinction of EYFP-positive and -negative subsets after treatment for BrdU staining, and EYFP^+^B220^+^ (IgA^+^ or IgA^−^) and B220^−^ subsets were analyzed. **(B)** Representative FACS profile of the fraction of BrdU-positive cells in the different fractions identified in A. **(C)** Analysis of the BrdU^+^ fraction from 4 mice, with the different subsets of each individual mouse represented by the same symbol. Mean values are represented. The data shown correspond to one experiment out of two performed in similar conditions. **, P < 0.01, one-way ANOVA with Holm-Sidak correction.

8 d of BrdU labeling was also performed, 2 mo after tamoxifen feeding, and EYFP^+^ cells were enriched by sorting, a procedure that allowed unambiguous identification of the lower level of EYFP fluorescence induced by the treatments required for BrdU staining (Fig. 8 A). A fraction of BrdU-labeled B cells was identified in the splenic EYFP^+^ compartment, for both plasma cells and memory B cells (16 and 26% for IgA^−^ and IgA^+^ memory B cells, respectively, the former population being essentially IgM^+^, and 27.5% for plasma cells; Fig. 8, B and C).

These two approaches clearly support the notion that constant input of persistent clones derived from mucosal immune reactions still feed the IgM and IgA peripheral pool several months after their initial AID-dependent labeling.

### Spleen EYFP^+^ IgM^+^ B cells show reactivity against commensal and infectious bacterial strains

To study the specificities of the IgM memory compartment, we took advantage of the two-step culture system of Nojima et al., which allows B cell proliferation with acquisition of a GC phenotype and, if pursued in presence of IL-21, plasma cell differentiation (Nojima et al., 2011). 10-d cultures were used to test the specificities of secreted Ig present in the culture supernatant from spleen naive and EYFP^+^ B cells (50,000 sorted cells). Short-term cultures (4 d with IL-4) were used to expand and activate EYFP^+^ cells for generating hybridomas. Two different cultures were made from two conventional mice, and used at both time points for supernatant analysis and hybridoma generation, with two hybridoma fusions performed independently. Three different cultures from three axenic mice were pooled in a single fusion, and two of them further maintained for collecting supernatants. 38 hybridomas stably secreting IgM were generated from EYFP^+^ B cells of conventional mice, and 37 from germ-free mice.

Supernatants from bulk cultures or from individual hybridomas were tested for their reactivity against luminal antigens (a global extract of intestinal content, including food and bacterial antigens), against defined human bacterial isolates representing equivalents of mouse commensal, opportunistic, and pathogenic species, as well as against mouse ERVs (isolated from the supernatant of the Baki-1 cell line; Yu et al., 2012).

**Figure 9.**
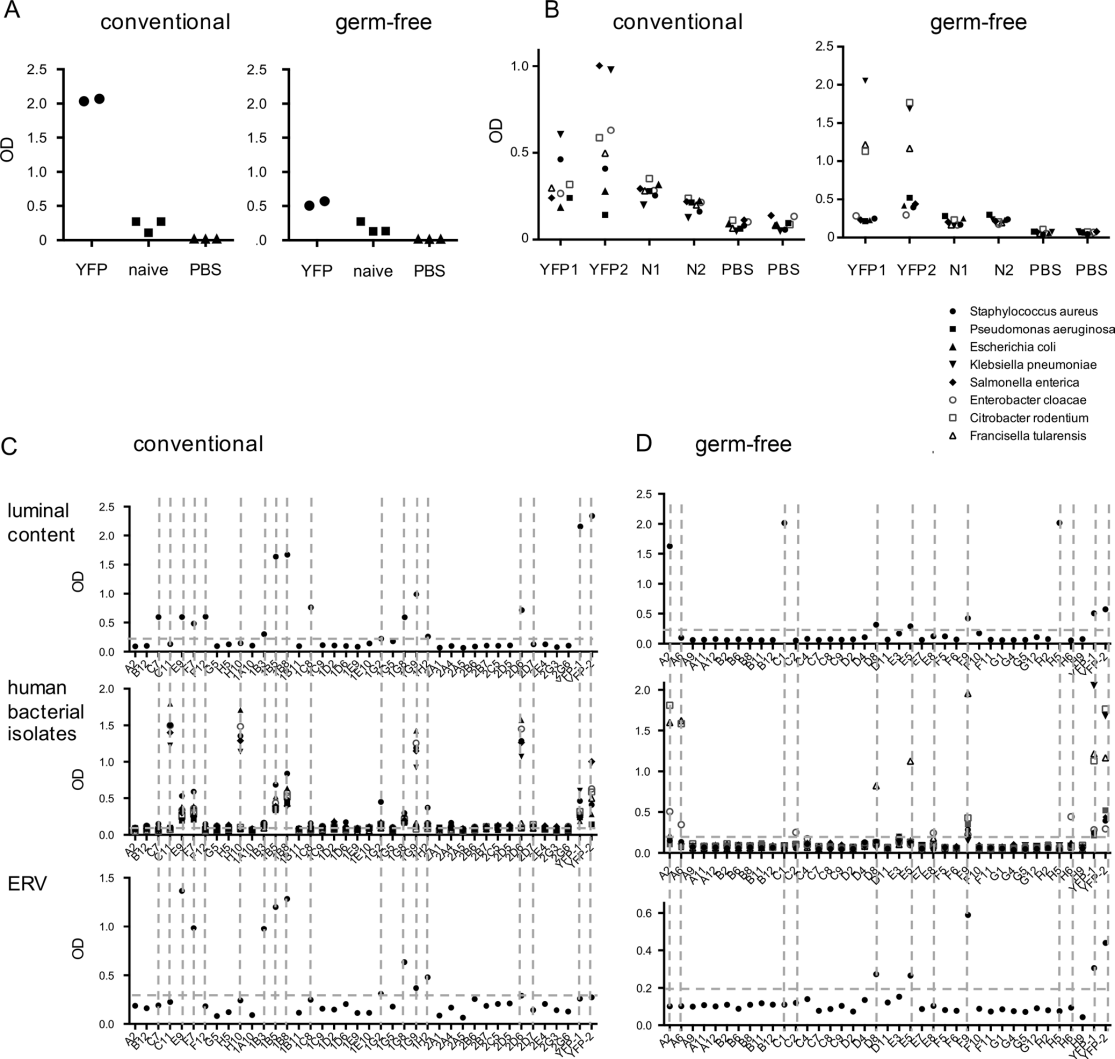
**Specificity of IgM memory B cells from conventional and germ-free mice.** The specificity of EYFP^+^ IgM memory B cells was assayed from bulk culture supernatants from either in vitro differentiated B cells or from individual hybridomas. **(A)** EYFP^+^IgM^+^ B cells from either conventional or germ-free mice were activated in vitro in the culture conditions of Nojima et al. (2011) (in presence of CD40L, BAFF, IL4 and IL21) and the supernatant tested by ELISA at day 10 against the gut luminal content, with supernatants from naive B cells as control. **(B)** The same supernatants (N1 and N2: naive B cells) were tested against a panel of eight human bacterial isolates, representing equivalents of mouse commensals or pathogens. **(C and D)** Hybridoma supernatants obtained from fusions performed on similar cultures at day 4 after activation of EYFP^+^ cells were tested by ELISA against gut luminal content, human bacterial isolates (same bacterial panel as in B) and supernatants from the ERV-producing Baki1 cell line. **(C)** Conventional mice. **(D)** Germ-free mice. The horizontal line marks background signal. YFP-1 and YFP-2 correspond to supernatants from day 10 cultures. Two different fusions were performed for EYFP^+^ B cells from two different conventional mice and one fusion from three germ-free mice.

Strong reactivity was observed against luminal antigens for supernatants from conventional mice and much less so for germ-free mice (Fig. 9 A). The profile of bacterial antigen recognition differed between conventional and germ-free mice, a pattern confirmed at the level of individual hybridomas (Fig. 9 B). Reactivity against endogenous retroviral antigens was altogether low (shown as controls in Fig. 9 C).

Hybridomas showed different patterns of bacterial antigen recognition, linked or not with positivity for luminal antigens (Fig. 9 C). Several cases of reactivity against ERVs were also observed, but always cosegregated with a positive bacteria or luminal reaction. Recognition of multiple bacterial isolates suggested that common bacterial structures may be targeted. Moreover, hybridoma supernatants that recognized the eight bacterial species tested were also reactive against luminal content and ERVs, which could correspond to the recognition of conserved glycosylated motifs (e.g., E9). Interestingly, similar bacterial patterns could be observed that mobilized different V_H_ genes (e.g., E9 and 1B5 from conventional mice, see Table S2) or the same V_H_ with different junctions (e.g., H10 and 1G9) and originated in both cases from different animals. Altogether, 15 out of 38 hybridomas from conventional mice showed positivity against the selected set of antigens (40%), and 10 out of 37 (27%) for germ-free mice (Fig. 9, C and D).

V_H_ gene usage, N addition, CDR3 length, and mutation load were determined for each hybridoma with positive reactivity and listed in Table S2. The V_H_6-3 gene (J606 family) was frequently represented, with various junctions and mutation load. Six cases of identical clones, most likely generated by in vitro clonal expansion, were observed, which accordingly displayed similar recognition profile. The mutation load clearly differed between hybridomas from conventional and germ-free, while it was globally higher than in the J_H_4 intron sequence, possibly suggesting positive selection for mutation in functional V_H_ genes.

Interestingly, bacterial recognition was shifted toward infectious species (*Citrobacter rodentium* and *Francisella tularensis*) in hybridomas from germ-free mice (Fig. 9 D). This reactivity was mediated by different V_H_ genes, including V_H_6-3, which, in A6 hybridoma, was unmutated.

Supernatants from bulk cultures were also tested for their reactivity against autoantigens, using the autoantigen protein array at Southwestern University, Texas, and, apart from dsDNA, did not show consistent autoantigen recognition, and did not perform significantly better than supernatants from B1 cells (data not shown). Hybridoma supernatants were also tested for polyreactivity, and no hybridoma showed corecognition of insulin and dsDNA (data not shown).

### Early response of CD73^+^CD80^+^ B cells to bacterial challenge and protective capacity of their secreted IgM products

**Figure 10.**
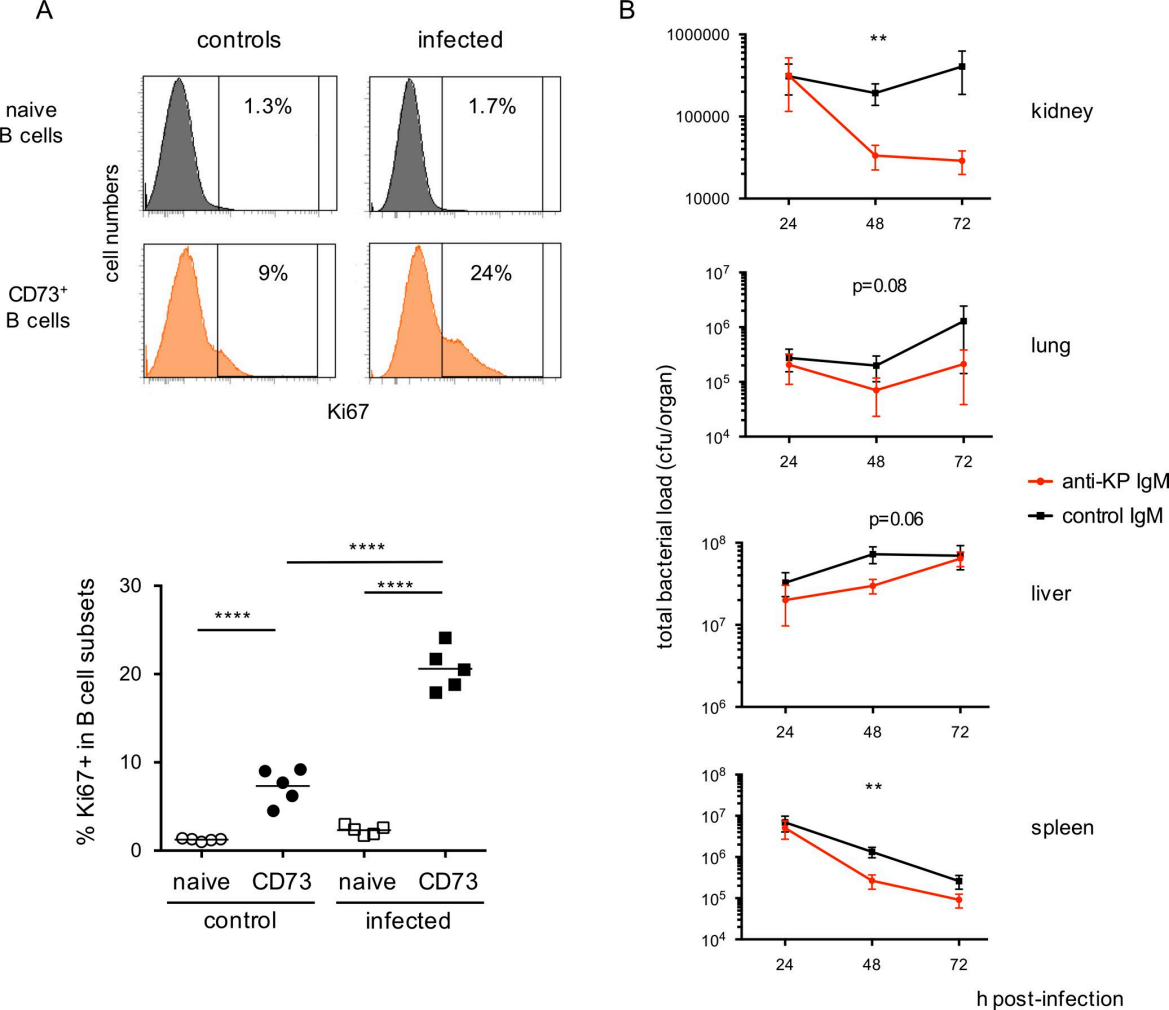
**Early response of the IgM^+^CD73^+^ subset to bacterial challenge. (A)** Mice were challenged with i.v. injection of 10^7^ *K. pneumoniae*, and Ki67 labeling of spleen cells was performed 3 d after and compared with noninfected mice (controls). Top part, representative FACS profile of Ki67 labeling percentage among naive and total CD73^+^ B cells (B220^+^-gated) from infected mice and controls. Bottom part, Ki67 labeling percentage among total naive and CD73^+^ spleen B cells from *K. pneumoniae*–infected or control mice. ****, P < 0.0001, one-way ANOVA with Holm-Sidak correction. Mean values are indicated. Results shown correspond to two different experiments. **(B)** Mice were injected i.p. with purified IgM from C11 and 1G9 hybridomas (500 µg each, with anti–*K. pneumoniae* reactivity, see Fig. 9 C) or from 1E9 and 2G3 hybridomas as control. 10^7^ *K. pneumoniae* were injected i.p. 1 h later, and bacterial load followed over 72 h in kidney, lung, spleen, and liver. Five animals were analyzed in each group for each time point. Animals from two different cohorts were pooled for analysis. Mean values ± SEM are indicated. **, P < 0.01, Mann-Whitney test.

Reactivity against *Klebsiella pneumoniae* was observed in culture supernatants from both germ-free and conventional mice. We thus assessed whether CD73^+^CD80^+^ B cells would show specific mobilization upon inoculation of these bacteria. To avoid differentiation into GC or plasma cells that would alter their surface phenotype and compromise their identification, we focused on the proliferation induced 3 d after bacterial injection (10^7^ bacteria, intravenous injection). We used only CD73 as marker, because CD80 was not compatible with Ki67 staining. All mice survived the infection, and CD73^+^ B cells showed an increased frequency of Ki67 labeling, compared with control mice (20.6 vs. 7.3%, Fig. 10, A and B) and to naive B cells from the same animals (1.2 and 2.3%; Fig. 10, A and B). The CD73^+^ compartment thus appears as an early responder during bacterial infection.

To test more directly the functional role of the IgM subset, we assessed the protective capacity of IgM secreted from bacteria-reactive hybridoma against the same *K. pneumoniae* isolate. We injected purified IgM from two different hybridomas with similar recognition profile (500 µg of each C11 and 1G9 hybridomas, i.p.) or from hybridomas without reactivity against these bacteria (2G3 and 1E9). The bacterial load was followed over 72 h in lung, kidney, liver, and spleen, with 10 mice analyzed at each time point in both settings. A significant difference in spleen and kidney was observed at 48 h between the groups injected with the bacteria-reactive antibodies or with antibody controls, with a reduction in bacterial load paralleling the overall level of infection. The bacterial load was also reduced in lung and liver, but the difference observed failed to reach significance (p-values of 0.08 and 0.06 in lung and liver, respectively). The IgM memory pool thus harbors antigenic specificities that can, to some extent, impact infection with a pathogen through recognition of gut microbiota cross-reactive epitopes.

## Discussion

We report here the description of a systemic memory population which is revealed during a 2-wk labeling period through its EYFP expression triggered by a tamoxifen-inducible Cre recombinase controlled by the *Aicda* promoter (Dogan et al., 2009). This B cell population was observed in the absence of deliberate immunization, persisted over 1 yr and displayed mutated Ig genes. It consists in spleen of a major IgM memory subset, including also IgA memory B cells, as well as a smaller subset of IgA and IgM plasma cells. In BM, IgA plasma cells predominate, together with a smaller subset of IgM-secreting cells.

AID-dependent labeling was acquired during activation in spontaneous GC in spleen, PPs, and MLNs. Rather remarkably, EYFP labeling of GC B cells remained for up to one year in PPs, while it waned in the spleen after 2 wk. Persistence of the EYFP^+^ splenic population appeared surprisingly to be achieved, not through its specific longevity, but through dynamic replenishment from B cell clones persisting in GCs from PPs. This was demonstrated by three convergent approaches: first, by Ig gene sequencing performed on different EYFP^+^ B cell subsets which allowed the identification of clonal relationships between cells from different tissues (PPs, spleen, and BM), isotypes (IgM and IgA), and differentiation stage (memory B cells and plasma cells); second, by BrdU labeling experiments, which showed a constant input of EYFP-marked, B cells in the spleen; and finally by anti-CD20 mediated B cell depletion, which put in evidence, upon termination of drug activity, the reconstitution of the splenic memory pool from PP GC EYFP^+^ B cells that escaped deletion.

All AID-labeled cells harbored a CD73^+^CD80^+^ phenotype, markers that have been linked previously with a mature memory phenotype (Taylor et al., 2012; Zuccarino-Catania et al., 2014), together with other activation/memory markers like CD43, TACI, TIM-1, PD-L1, and, more heterogeneously, PD-L2, as well as “resident-type” integrins, like CD49f (Itga6) and CD29 (Itgb1). We therefore used these CD73^+^CD80^+^ markers to estimate the size of the total IgM^+^ memory subset in the mouse spleen. The IgM^+^CD73^+^CD80^+^ population reaches a plateau around 5 mo of age to ∼4% of the total splenic B cell pool and displays a mutation load comparable to the B220^+^EYFP^+^ subset. This CD73^+^CD80^+^ subset was markedly impacted in T cell–deficient animals, and also reduced in MyD88xTrif and TLR7-deficient mice. It should be stressed, however, that while our EYFP labeling procedure allows us to follow cell populations that are long-lasting either by being long-lived or renewed from persistent clones, additional immune reactions clearly contribute to the splenic C73^+^CD80^+^ IgM^+^ B cell pool without displaying such features (Fig. S5). Spontaneous splenic GCs have been previously described by the group of Rahman, who showed that they depended on IFNγ–IFNγR and TLR7 signaling and produced essentially IgG2b and IgG2c (Soni et al., 2014; Domeier et al., 2016). The plateau observed for the CD73^+^CD80^+^ population is thus likely to involve a global maintenance of the pool, with constant input and cell death from GALT-derived persistent clones, but also newly activated ones from spontaneous splenic GC, which fail to show clonal persistence. Some of the phenotypic differences observed between the EYFP^+^ and EYFP^−^ CD73^+^CD80^+^ splenic compartment (for, e.g., CD43 or PD-L2 expression) could correspond to the mixed origin of the splenic EYFP^−^ IgM memory pool.

Our study extends to the systemic immune system the observation of Pabst et al. of the longitudinal persistence of B cell clones feeding mucosal plasma cells from ongoing PP immune responses (Lindner et al., 2015). It clearly documents the contribution of such mucosal-derived effector B cells comprising large expanded clones, not only to the peripheral IgA plasma cell pool, but also to memory B cells including a major splenic IgM population. The IgM compartment is still present, albeit reduced, in germ-free mice, while both IgA memory and plasma cells are drastically affected. Dependence of the BM IgA plasma cell pool upon the gut flora has been recently reported by Wilmore et al. (2018). IgM memory B cells observed in the spleen of germ-free animals display a lower mutation load, suggesting that residual antigen activation in the gut, possibly through food antigens or endogenous triggers like ERVs, may not sustain mutation accumulation.

The antigen specificity of the spleen IgM^+^EYFP^+^ compartment, studied from supernatants of in vitro B cell cultures, as well as through the establishment of hybridomas, showed a broad cross-reactivity against antigens from the gut luminal content (which includes both commensals and food antigens), against multiple types of bacteria, as well as against ERVs. This indicates that recognition for common bacterial motifs, possibly glycan epitopes (Patel and Kearney, 2016), are preferentially selected in the systemic IgM memory pool. It should be noted accordingly that no unique reactivity against ERVs was observed, as it was always linked with bacterial or luminal content recognition, further documenting the cross-reactivity of these IgM antibodies. Interestingly, TLR7 is also a key element of the immune response against ERVs and bacterial products are important drivers of their expression (Yu et al., 2012; Young et al., 2014). A large fraction of hybridomas from germ-free mice still showed reactivity against these three categories of antigens, with a different profile for bacterial isolates, notably a preferential recognition of pathogenic species like *Francisella tularensis*. Moreover, due to the much lower mutation frequency observed in germ-free conditions, bacterial recognition was achieved in some cases by unmutated V_H_ genes (e.g., V6.3 of the J606 family), thus revealing new cases of germline-encoded antibacterial reactivity in the V gene repertoire.

Natural IgMs have multiple roles in immune homeostasis, from scavengers to protection against autoimmune processes (Ehrenstein and Notley, 2010). Their critical role in infectious processes has also been reported (Ochsenbein et al., 1999). B1 cells are important contributors of these spontaneously arising antibodies, but they are clearly not the sole source of circulating IgMs (Thurnheer et al., 2003; Reynolds et al., 2015). The monitoring of somatic mutation at the protein level has revealed that mutated IgMs accumulated with age in nonimmunized animals (Williams et al., 2000), an observation that clearly matches our description of a mutated IgM memory subset in the absence of external challenge. The broad antibacterial recognition profile identified within this subset suggests that, in addition to B1 B cell–derived antibodies, mutated IgM antibodies may represent an additional line of defense, affording a protection against systemic microbial translocation, a process that takes place in both healthy and pathological contexts (Brenchley and Douek, 2012), or external bacterial aggressions.

This work thus describes a new layer of B cell memory, maintained through ongoing renewal of persistent clones diversifying in the gut and thereafter reaching the systemic immune compartment, where they can be activated by cognate or cross-reactive antigens. It has been recently proposed that the gut microbiota may also shape the preimmune repertoire (Chen et al., 2018). Nevertheless, the memory IgM subset we describe clearly stands out as a more rapid responder to bacterial challenge, compared with naive B cells.

IgM memory B cells have emerged as integral effectors of recall immune responses in the mouse (Dogan et al., 2009; Zuccarino-Catania et al., 2014; Krishnamurty et al., 2016). Mutated IgM B cells represent a large subset in humans as well, but their origin, function, and diversification is still a matter of debate. It will be obviously not possible to identify a pool of activated B cells generated in the absence of external challenge, but the contribution of IgM B cell clones activated in the gut to the peripheral B cell repertoire is a more testable question, and recognition of common bacterial epitopes has already been suggested for some human memory subsets, like marginal zone or CD27^−^IgD^−^ B cells (Puga et al., 2012; Berkowska et al., 2015). Very recently, Wardemann et al. described in human blood the presence of IgM memory B cells specific for *K. pneumoniae* LPS glycan antigens, cells that harbor clonal relationships with plasma cells from the lamina propria and display cross-specificity for various bacterial commensal species as well as for HIV gp140 glycoprotein (Rollenske et al., 2018). This study deepens the link previously reported between reactivity against epitopes of the gut flora and antiviral responses (Trama et al., 2014; Williams et al., 2015), and strongly supports the proposition of a protective role afforded by systemic IgM memory B cells recognizing epitopes shared between mucosal symbionts, pathobionts, and overt pathogens.

## Materials and methods

### Mouse lines

Heterozygous AID reporter mice were generated by breeding AID-Cre-ERT2 mice with the ROSA26-loxP-EYFP reporter mice (Dogan et al., 2009) and are named “AID-Cre-EYFP” throughout this paper. The *Aicda* and *ROSA26* loci are tightly linked on chromosome 6, and the line used was isolated from a recombination event that brought both reporters on the same chromosome.

MyD88xTrif-deficient mice were kindly given by Catherine Werts and Ivo Gomperts Boneca (Institut Pasteur, Paris, France), *CD3ε* KO mice by Sophie Ezine (INEM, Paris, France), and TLR7-deficient mice by Isabelle Cremer (Centre de Recherche des Cordeliers, Paris, France). All gene-targeted mice were analyzed between 5 and 6 mo of age. AID-Cre-EYFP mice were rendered axenic by cesarean transfer at the CDTA (Orléans, France) and checked regularly for lack of bacterial colonization (under the supervision of Alexandre Diet, coordinator, and Cécile Frémond, veterinary).

All mouse experimental protocols have been approved by the Paris Descartes Ethics Committee and authorized by the French Ministery of Research.

### Generation of the BAC AID-Cre-ERT2 mouse line

To avoid *Aicda* haploinsufficiency linked with targeted insertion of the Cre-ERT2 gene at the *Aicda* locus, we generated a line in which the same targeting event was achieved within a bacterial artificial chromosome. DNA from AID-Cre-ERT2 mice was used to amplify a 6-kb Cre-ERT2 fragment flanked with 2-kb sequences from the *Aicda* locus, cloned in vector pLD53 (gift of Nina Papavassiliou, Rockefeller University, New York, NY) and transformed into PIR2 One Shot competent bacteria (Invitrogen). The RP24-68I7 BAC strain (sequence NCBI AC158651) that include 190 kb of the *Aicda* locus was obtained from BACPAC resources (Children’s Hospital Oakland Research Institute, Oakland, CA) and the pSV1-RecA vector containing the AID-Cre-ERT2 insert vector was used to promote homologous recombination by successive selection steps according to (Misulovin et al., 2001). The BAC DNA was extracted according to the Transgenic Animal Web protocol and microinjected into C57BL/6 fertilized eggs. Two independent mouse lines containing each a single BAC copy were obtained and gave similar EYFP-labeling profiles.

### Tamoxifen regimen

Doses of 10 mg Nolvadex (AstraZeneca) in 500 µl of 20% Clinoleic (Baxter) were administered by gavage every 5 d for three times (day 0, 5, and 10). Tamoxifen experiments with young versus adult mice were done with a single dose of tamoxifen. Here the amount of tamoxifen was adjusted to the weight of animal for young mice. Axenic mice received an irradiated tamoxifen diet during 2 wk (400 mg tamoxifen citrate/kg diet; TD55125I; Harlan Laboratories Europe), and control mice were fed similarly.

### Confocal microscopy

8-µm cryosections were prepared from spleen and PPs fixed with 4% paraformaldehyde (PFA) and dehydrated overnight in 30% sucrose before freezing in optimal cutting temperature compound. For immunostaining, samples were saturated with PBS supplemented with 10% (vol/vol) goat serum, labeled with primary antibodies and secondary reagents (listed in Table S3) in PBS supplemented with 0.5% BSA, mounted with Fluoromount (Southern Biotechnology Associates) and analyzed with a Leica TCS SP8 STED confocal microscope.

### Flow cytometry analysis and cell sorting

Splenic populations were separated using a FACSAria cell sorter (BD Biosciences) for cell culture experiments. Splenic subsets were also sorted for the analysis of mutations in the rearranged J_H_4 intronic sequences of the IgH locus. For flow cytometry analysis, cell suspensions of spleen, PPs, lymph nodes, and other tissues were incubated on ice for 20 min with combinations of fluorochrome-conjugated antibodies in PBS supplemented with 0.5% BSA. Red blood cells from lymphoid tissues (spleen and BM) were lysed before staining. When necessary, cells were stained by PeCy7-conjugated streptavidin as secondary reagent. Antibodies used in this study are summarized in Table S3. Dead cells were excluded by using 7-aminoactinomycin D staining in the analysis. For intracellular isotypes and Blimp-1 staining, cells were first stained for surface antigens, then fixed, and permeabilized with Cytofix/Cytoperm solution (BD Biosciences). For Ki67 labeling, cells were fixed in 4% PFA (20 min at 4°C) and permeabilized in 0.5% Triton X-100 (20 min, room temperature). Cells were then washed and stained for Ki67 in 0.5% Triton X-100. After washing, cells were resuspended in PBS containing 2% FCS and collected on BDLSR Fortessa apparatus. Analyses were done with FlowJo (Tree Star Inc.) or Diva (Becton Dickinson) softwares.

### Adoptive cell transfer

3 × 10^6^ total cells from peritoneal cavity as source of B-1 cells and 3 × 10^6^ total cells from BM as source of B-2 cells in 200 µl PBS were injected intravenously in recipient mice that have been sublethally irradiated with 8.5 Gy 12 h before, according to the protocol described in Baumgarth et al. (1999). Recipient mice received three doses of tamoxifen 6 wk after cell injection and were analyzed 8 wk after the last tamoxifen.

### In vitro B cell cultures and supernatant collection

Purified splenic naive B cells and splenic EYFP^+^ memory B cells (5 × 10^4^ cells per well; Nojima et al., 2011) were cultured in 6-well plates in the presence of 3T3 40LB cells (3 × 10^5^ cells per well) that had been pretreated with mitomycin C (1 mg/ml during 3 h; Sigma) in 8 ml RPMI-1640 medium supplemented with 10% FCS, 5.5 × 10^−5^ M 2-mercaptoethanol, 10 mM Hepes, 1 mM sodium pyruvate, 100 U/ml penicillin, and 100 µg/ml streptomycin (GIBCO). rIL-4 (1 ng/ml; Peprotech) was added to the primary culture for 4 d. On day 4, the cells were replated (5 × 10^5^ cells) onto a new feeder layer treated with mitomycin C (3 × 10^6^ cells) in a 10-cm tissue culture dish and cultured in 40 ml of medium supplemented with rIL-21 (10 ng/ml, Peprotech) for 6 d. Cells were cultured in a humidified atmosphere at 37°C with 5% CO_2_. At day 10, 38 ml of supernatant were collected and concentrated using an Amicon Ultra 100000 MCWO (Millipore).

### EYFP Hybridomas generation

5 × 10^4^ sorted splenic memory EYFP^+^ B cells were amplified during 4 d on the 3T3 40LB feeder layer in presence of IL-4. EYFP^+^ cells were added to Sp2/0 myeloma cell line in a ratio 1:1. Mixed cells were washed three times in DMEM and then cells were fused using the ClonaCell-HY hybridoma kit (StemCell Technologies, Inc.; Le Gallou et al., 2017).

### Analysis of mutations in the J_H_4 intronic sequence of the IgH locus and rearranged V_H_ genes of hybridomas 

The J_H_4 intronic sequence flanking rearranged V_H_ gene segments was amplified by PCR from DNA of sorted B cell subsets, from 10,000 to 1,000 cells. The PCR primers used are in 5′, a mixture of eight FR3 primers amplifying most V_H_ gene families (V_H_1: 5′-GAGGACTCTGCRGTCTATTWCTG-3′, V_H_3: 5′-GAGGACACACCCACATATTACTG-3′, V_H_5: 5′-GAGGACACRGCCATGTATTACTG-3′, V_H_6: 5′-GAAGACACTGGAATTTATTACTG-3′, V_H_7: 5′-GAGGACAGTGCCACTTATTACTG-3′, V_H_9: 5′-ATGAGGACATGGCTACATATTTC-3′, V_H_10/12: 5′-GAGGACACAGCCATGTATTACTG-3′, V_H_11: 5′-GAGGACACAGCCACGTATTTCTG-5′, in a 6:1:3:1:1:1:1:1 ratio); in 3′, a J_H_4 intronic primer (J_H_4rev: 5′-CACCAGACCTCTCTAGACAGC-3′), with a nested amplification performed in cases of low cell numbers (using J_H_4-nested: 5′-TGAGACCGAGGCTAGATGCC-3′ and the same V_H_ primer set; 2 min at 98°C and 40–50 cycles of 15 s at 98°C, 30 s at 64°C and 30 s at 72°C, with Phusion DNA polymerase [New England Biolabs]; and 30 additional cycles in cases of nested PCR). PCR products were cloned with the Zero Blunt cloning kit (Invitrogen) and sequences were determined with an ABI Prism 3130xl Genetic Analyzer. Mutations were identified within 448 bp of the J_H_4 intron and analyzed with the help of the CodonCodeAligner software. Between 25 and 96 sequences per sample were determined for mutation frequency determination. Clonal relationships were established using CodonCodeAligner, plus manual inspection, notably to discard clonal relationships that would include sequence changes at the V-D or D-J junction.

For hybridoma sequences, total RNA was isolated with the RNeasy Micro kit (QIAGEN) and reverse transcribed by random priming with the Affinityscript Multiple Temperature cDNA synthesis kit (Agilent Technologies). The following primers amplifying V_H_1, V_H_3, V_H_5, V_H_11 and V_H_12 families (in a 5:1.5:1.5:1:1 ratio), together with a reverse Cµ primer were used for amplification: mV_H_1, 5′-AGGTYCAGCTGCARCAGTCT-3′; mV_H_3, 5′-GTGCAGCTTCAGGAGTCAG-3′; mV_H_5, 5′-GAAGTGAAGCTGGTGGAGTC-3′; mV_H_11, 5′-ATGGAGTGGGAACTGATCTTA-3′; mV_H_12, 5′-AGCTTCAGGAGTCAGGACC-3′; Cµ, 5′-CATGGCCACCAGATTCTTATC-3′. PCR amplification conditions were 94°C 45 s, 62°C 1 min, and 72°C 1.5 min for 35 cycles with GoTaq polymerase (Promega), and 20 pmol of primers. PCR products were gel-purified and 50 ng were sequenced with the Cµ primer.

### BrdU labeling

Cell proliferation was assessed 2–3 mo after tamoxifen labeling. BrdU was given at 0.8 mg/ml in the drinking water for 8 d, with water supply changed every day. Splenic cell suspensions were stained with the desired antibodies (B220, IgA, GL7) and the LIVE/DEAD Fixable Aqua Amcyan Dead Cell Stain kit was used for gating out dead cells. Fixation with cytofix/cytoperm buffer (BrdU APC kit, Beckton Dickinson) was performed before enrichment of EYFP^+^ cells by cell sorting. This enrichment procedure allows the correct identification of EYFP^+^ cells after the BrdU staining procedure that lowers the EYFP signal, while keeping sufficiently EYFP^−^ cells for internal control. BrdU staining was performed on sorted cells (8–15 × 10^4^ cells) after fixation and permeabilization using the BrdU APC kit, according to the conditions of the supplier, with adaptation of the protocol for low cell numbers (anti-BrdU-APC antibody diluted 1/150e in 50 µl for ∼10 × 10^4^cells).

### Anti-CD20 depletion

B cell depletion was performed by three injections, 5 d apart, of the 18B12 anti-CD20 antibody, 1 mo after tamoxifen labeling (250 µg per injection, i.p., mouse IgG2a, Biogen Idec). PP and spleen lymphocytes were analyzed at day 50 (nadir) or day 90–107. The efficiency of B cell depletion was checked on blood for all animals at day 50.

### ELISA

Antibody concentrations in supernatants were determined by anti-mouse IgM ELISA using mouse monoclonal IgM as standard (Sigma). Supernatants were used at 1 µg/ml. For detection of IgM against bacteria, bacteria from human clinical isolates were prepared by overnight cultures on LB agar Petri dishes. 10 µl was harvested using a sterile inoculating loop and resuspended in 1 ml of PBS. Lysis of bacteria was performed in a FastPrep instrument (three runs of 45 s at a setting of 6), followed by centrifugation (5 min at 12,000 *g*). To ensure that no live bacteria remained, pellets were resuspended in PBS and incubated 30 min at RT with 10 µg/ml of polymyxin B (for *Pseudomonas aeruginosa*, *Escherichia coli*, *K*. *pneumoniae*, *Salmonella enterica*, *Enterobacter cloacae*, *Citrobacter rodentium*, and *Francisella tularensis*) or vancomycin (*Staphylococcus aureus*) before plating aliquots on LB agar. Killed bacteria were coated at 5 µg/ml. ELISAs were performed as above and OD was read at 450 nm.

For detection of antiendogenous retroviral specificities, ERVs purified from supernatants of Baki-1 cell line according to Yu et al. (2012), were used at 5 µg/ml in PBS for coating in 96-well plates.

### In vivo infection

*K. pneumoniae* (strain KP B13E6, used for hybridoma supernatant assays) was grown in LB to exponential growth phase and diluted to the appropriate concentrations in saline solution (0.9% wt/vol of NaCl). 10^7^ bacteria were injected i.v. for proliferation assays of CD73^+^ cells. To test for their protective capacity, IgM were purified from supernatants of hybridoma cultures performed in endotoxin-free conditions by ion exchange chromatography (RD Biotech). Two groups of 6–8-wk-old female BALB/c mice (Janvier) were pretreated with MAbs, 4 h before challenge: one group was injected i.p. with 1 ml of a 1/1 mixture of bacteria-reactive MAbs (hybridoma C11 and 1G9 at a final concentration of 1 mg/ml); and a control group was injected i.p. with 1 ml of a 1/1 mixture of control MAbs (hybridomas 2G3 and 1E9, 1 mg/ml final). Mice were then i.p. inoculated with 200 µl of a bacterial suspension containing 1.2 × 10^7^ CFUs per mouse. Inoculum titers were checked by spreading dilutions of the preparations on LB agar plates. Two groups of five mice were analyzed for each time point for each group, at 24, 48, and 72 h after infection. For bacterial burden determination, homogenized spleen, liver, lung, and kidney tissues were isolated from each individual mouse, serially diluted and spread on to LB agar plates.

### Statistics

Student’s two-tailed *t* tests or Mann-Whitney tests were used, as well as one-way ANOVA test with Holm-Sidak correction for multiple comparisons.

### Online supplemental material

Fig. S1 reports EYFP^+^ cell distribution in various tissues and EYFP-labeling efficiency of an alternate fate-mapping model. Fig. S2 shows confocal images of EYFP labeling in GCs. Fig. S3 shows EYFP labeling of B cell subsets in the peritoneal cavity. Fig. S4 shows clonal distribution of V_H_ sequences recovered after anti-CD20 depletion. Fig. S5 shows a schematic proposition of tissue contribution to the splenic IgM memory pool. Table S1 shows mutation frequencies for the CD73^+^CD80^+^EYFP^−^ IgM subset; Table S2 shows V_H_ characteristics of hybridoma sequences; and Table S3 shows antibody references.

## Supplementary Material

Supplemental Materials (PDF)

## References

[bib1] Baumgarth N., Herman O.C., Jager G.C., Brown L., Herzenberg L.A., and Herzenberg L.A. 1999 Innate and acquired humoral immunities to influenza virus are mediated by distinct arms of the immune system. Proc. Natl. Acad. Sci. USA. 96:2250–2255. 10.1073/pnas.96.5.2250 10051627 PMC26769

[bib2] Baumjohann D., Preite S., Reboldi A., Ronchi F., Ansel K.M., Lanzavecchia A., and Sallusto F. 2013 Persistent antigen and germinal center B cells sustain T follicular helper cell responses and phenotype. Immunity. 38:596–605. 10.1016/j.immuni.2012.11.020 23499493

[bib3] Belkaid Y., and Hand T.W. 2014 Role of the microbiota in immunity and inflammation. Cell. 157:121–141. 10.1016/j.cell.2014.03.011 24679531 PMC4056765

[bib4] Bemark M., Hazanov H., Strömberg A., Komban R., Holmqvist J., Köster S., Mattsson J., Sikora P., Mehr R., and Lycke N.Y. 2016 Limited clonal relatedness between gut IgA plasma cells and memory B cells after oral immunization. Nat. Commun. 7:12698 10.1038/ncomms12698 27596266 PMC5025876

[bib5] Berkowska M.A., Schickel J.N., Grosserichter-Wagener C., de Ridder D., Ng Y.S., van Dongen J.J., Meffre E., and van Zelm M.C. 2015 Circulating Human CD27-IgA+ Memory B Cells Recognize Bacteria with Polyreactive Igs. J. Immunol. 195:1417–1426. 10.4049/jimmunol.1402708 26150533 PMC4595932

[bib6] Brenchley J.M., and Douek D.C. 2012 Microbial translocation across the GI tract. Annu. Rev. Immunol. 30:149–173. 10.1146/annurev-immunol-020711-075001 22224779 PMC3513328

[bib7] Bunker J.J., Flynn T.M., Koval J.C., Shaw D.G., Meisel M., McDonald B.D., Ishizuka I.E., Dent A.L., Wilson P.C., Jabri B., 2015 Innate and Adaptive Humoral Responses Coat Distinct Commensal Bacteria with Immunoglobulin A. Immunity. 43:541–553. 10.1016/j.immuni.2015.08.007 26320660 PMC4575282

[bib8] Chen Y., Chaudhary N., Yang N., Granato A., Turner J.A., Howard S.L., Devereaux C., Zuo T., Shrestha A., Goel R.R., 2018 Microbial symbionts regulate the primary Ig repertoire. J. Exp. Med. 215:1397–1415. 10.1084/jem.20171761 29588346 PMC5940265

[bib9] Dogan I., Bertocci B., Vilmont V., Delbos F., Mégret J., Storck S., Reynaud C.A., and Weill J.C. 2009 Multiple layers of B cell memory with different effector functions. Nat. Immunol. 10:1292–1299. 10.1038/ni.1814 19855380

[bib10] Domeier P.P., Chodisetti S.B., Soni C., Schell S.L., Elias M.J., Wong E.B., Cooper T.K., Kitamura D., and Rahman Z.S. 2016 IFN-γ receptor and STAT1 signaling in B cells are central to spontaneous germinal center formation and autoimmunity. J. Exp. Med. 213:715–732. 10.1084/jem.20151722 27069112 PMC4854731

[bib11] Ehrenstein M.R., and Notley C.A. 2010 The importance of natural IgM: scavenger, protector and regulator. Nat. Rev. Immunol. 10:778–786. 10.1038/nri2849 20948548

[bib12] Gomez de Agüero M., Ganal-Vonarburg S.C., Fuhrer T., Rupp S., Uchimura Y., Li H., Steinert A., Heikenwalder M., Hapfelmeier S., Sauer U., 2016 The maternal microbiota drives early postnatal innate immune development. Science. 351:1296–1302. 10.1126/science.aad2571 26989247

[bib13] Hao Y., O’Neill P., Naradikian M.S., Scholz J.L., and Cancro M.P. 2011 A B-cell subset uniquely responsive to innate stimuli accumulates in aged mice. Blood. 118:1294–1304. 10.1182/blood-2011-01-330530 21562046 PMC3152496

[bib14] Hooper L.V., and Macpherson A.J. 2010 Immune adaptations that maintain homeostasis with the intestinal microbiota. Nat. Rev. Immunol. 10:159–169. 10.1038/nri2710 20182457

[bib15] Jarjour M., Jorquera A., Mondor I., Wienert S., Narang P., Coles M.C., Klauschen F., and Bajénoff M. 2014 Fate mapping reveals origin and dynamics of lymph node follicular dendritic cells. J. Exp. Med. 211:1109–1122. 10.1084/jem.20132409 24863064 PMC4042641

[bib16] Kim M., Qie Y., Park J., and Kim C.H. 2016 Gut Microbial Metabolites Fuel Host Antibody Responses. Cell Host Microbe. 20:202–214. 10.1016/j.chom.2016.07.001 27476413 PMC4982788

[bib17] Krishnamurty A.T., Thouvenel C.D., Portugal S., Keitany G.J., Kim K.S., Holder A., Crompton P.D., Rawlings D.J., and Pepper M. 2016 Somatically Hypermutated Plasmodium-Specific IgM(+) Memory B Cells Are Rapid, Plastic, Early Responders upon Malaria Rechallenge. Immunity. 45:402–414. 10.1016/j.immuni.2016.06.014 27473412 PMC5118370

[bib18] Kuraoka M., Holl T.M., Liao D., Womble M., Cain D.W., Reynolds A.E., and Kelsoe G. 2011 Activation-induced cytidine deaminase mediates central tolerance in B cells. Proc. Natl. Acad. Sci. USA. 108:11560–11565. 10.1073/pnas.1102571108 21700885 PMC3136303

[bib19] Lécuyer E., Rakotobe S., Lengliné-Garnier H., Lebreton C., Picard M., Juste C., Fritzen R., Eberl G., McCoy K.D., Macpherson A.J., 2014 Segmented filamentous bacterium uses secondary and tertiary lymphoid tissues to induce gut IgA and specific T helper 17 cell responses. Immunity. 40:608–620. 10.1016/j.immuni.2014.03.009 24745335

[bib20] Le Gallou S., Nojima T., Kitamura D., Weill J.C., and Reynaud C.A. 2017 The AID-Cre-ERT2 Model: A Tool for Monitoring B Cell Immune Responses and Generating Selective Hybridomas. Methods Mol. Biol. 1623:243–251. 10.1007/978-1-4939-7095-7_19 28589361

[bib21] Lemke A., Kraft M., Roth K., Riedel R., Lammerding D., and Hauser A.E. 2016 Long-lived plasma cells are generated in mucosal immune responses and contribute to the bone marrow plasma cell pool in mice. Mucosal Immunol. 9:83–97. 10.1038/mi.2015.38 25943272

[bib22] Lindner C., Thomsen I., Wahl B., Ugur M., Sethi M.K., Friedrichsen M., Smoczek A., Ott S., Baumann U., Suerbaum S., 2015 Diversification of memory B cells drives the continuous adaptation of secretory antibodies to gut microbiota. Nat. Immunol. 16:880–888. 10.1038/ni.3213 26147688

[bib23] Mahévas M., Patin P., Huetz F., Descatoire M., Cagnard N., Bole-Feysot C., Le Gallou S., Khellaf M., Fain O., Boutboul D., 2013 B cell depletion in immune thrombocytopenia reveals splenic long-lived plasma cells. J. Clin. Invest. 123:432–442. 10.1172/JCI65689 23241960 PMC3533302

[bib24] Mei H.E., Yoshida T., Sime W., Hiepe F., Thiele K., Manz R.A., Radbruch A., and Dörner T. 2009 Blood-borne human plasma cells in steady state are derived from mucosal immune responses. Blood. 113:2461–2469. 10.1182/blood-2008-04-153544 18987362

[bib25] Mei H.E., Frölich D., Giesecke C., Loddenkemper C., Reiter K., Schmidt S., Feist E., Daridon C., Tony H.P., Radbruch A., and Dörner T. 2010 Steady-state generation of mucosal IgA+ plasmablasts is not abrogated by B-cell depletion therapy with rituximab. Blood. 116:5181–5190. 10.1182/blood-2010-01-266536 20829370

[bib26] Misulovin Z., Yang X.W., Yu W., Heintz N., and Meffre E. 2001 A rapid method for targeted modification and screening of recombinant bacterial artificial chromosome. J. Immunol. Methods. 257:99–105. 10.1016/S0022-1759(01)00452-5 11687243

[bib27] Nojima T., Haniuda K., Moutai T., Matsudaira M., Mizokawa S., Shiratori I., Azuma T., and Kitamura D. 2011 In-vitro derived germinal centre B cells differentially generate memory B or plasma cells in vivo. Nat. Commun. 2:465 10.1038/ncomms1475 21897376

[bib28] Ochsenbein A.F., Fehr T., Lutz C., Suter M., Brombacher F., Hengartner H., and Zinkernagel R.M. 1999 Control of early viral and bacterial distribution and disease by natural antibodies. Science. 286:2156–2159. 10.1126/science.286.5447.2156 10591647

[bib29] Palm N.W., de Zoete M.R., Cullen T.W., Barry N.A., Stefanowski J., Hao L., Degnan P.H., Hu J., Peter I., Zhang W., 2014 Immunoglobulin A coating identifies colitogenic bacteria in inflammatory bowel disease. Cell. 158:1000–1010. 10.1016/j.cell.2014.08.006 25171403 PMC4174347

[bib30] Patel P., and Kearney J.F. 2016 Immunological Outcomes of Antibody Binding to Glycans Shared between Microorganisms and Mammals. J. Immunol. 197:4201–4209. 10.4049/jimmunol.1600872 27864551 PMC5119654

[bib31] Proietti M., Cornacchione V., Rezzonico Jost T., Romagnani A., Faliti C.E., Perruzza L., Rigoni R., Radaelli E., Caprioli F., Preziuso S., 2014 ATP-gated ionotropic P2X7 receptor controls follicular T helper cell numbers in Peyer’s patches to promote host-microbiota mutualism. Immunity. 41:789–801. 10.1016/j.immuni.2014.10.010 25464855

[bib32] Puga I., Cols M., Barra C.M., He B., Cassis L., Gentile M., Comerma L., Chorny A., Shan M., Xu W., 2012 B cell-helper neutrophils stimulate the diversification and production of immunoglobulin in the marginal zone of the spleen. Nat. Immunol. 13:170–180. 10.1038/ni.2194 PMC3262910 22197976

[bib33] Reynolds A.E., Kuraoka M., and Kelsoe G. 2015 Natural IgM is produced by CD5- plasma cells that occupy a distinct survival niche in bone marrow. J. Immunol. 194:231–242. 10.4049/jimmunol.1401203 25429072 PMC4272881

[bib34] Rollenske T., Szijarto V., Lukasiewicz J., Guachalla L.M., Stojkovic K., Hartl K., Stulik L., Kocher S., Lasitschka F., Al-Saeedi M., 2018 Cross-specificity of protective human antibodies against Klebsiella pneumoniae LPS O-antigen. Nat. Immunol. 19:617–624. 10.1038/s41590-018-0106-2 29760533

[bib35] Rubtsov A.V., Rubtsova K., Fischer A., Meehan R.T., Gillis J.Z., Kappler J.W., and Marrack P. 2011 Toll-like receptor 7 (TLR7)-driven accumulation of a novel CD11c^+^ B-cell population is important for the development of autoimmunity. Blood. 118:1305–1315. 10.1182/blood-2011-01-331462 21543762 PMC3152497

[bib36] Song J., Lokmic Z., Lämmermann T., Rolf J., Wu C., Zhang X., Hallmann R., Hannocks M.J., Horn N., Ruegg M.A., 2013 Extracellular matrix of secondary lymphoid organs impacts on B-cell fate and survival. Proc. Natl. Acad. Sci. USA. 110:E2915–E2924. 10.1073/pnas.1218131110 23847204 PMC3732919

[bib37] Soni C., Wong E.B., Domeier P.P., Khan T.N., Satoh T., Akira S., and Rahman Z.S. 2014 B cell-intrinsic TLR7 signaling is essential for the development of spontaneous germinal centers. J. Immunol. 193:4400–4414. 10.4049/jimmunol.1401720 25252960 PMC4201954

[bib38] Tas J.M., Mesin L., Pasqual G., Targ S., Jacobsen J.T., Mano Y.M., Chen C.S., Weill J.C., Reynaud C.A., Browne E.P., 2016 Visualizing antibody affinity maturation in germinal centers. Science. 351:1048–1054. 10.1126/science.aad3439 26912368 PMC4938154

[bib39] Taylor J.J., Pape K.A., and Jenkins M.K. 2012 A germinal center-independent pathway generates unswitched memory B cells early in the primary response. J. Exp. Med. 209:597–606. 10.1084/jem.20111696 22370719 PMC3302224

[bib40] Thurnheer M.C., Zuercher A.W., Cebra J.J., and Bos N.A. 2003 B1 cells contribute to serum IgM, but not to intestinal IgA, production in gnotobiotic Ig allotype chimeric mice. J. Immunol. 170:4564–4571. 10.4049/jimmunol.170.9.4564 12707334

[bib41] Trama A.M., Moody M.A., Alam S.M., Jaeger F.H., Lockwood B., Parks R., Lloyd K.E., Stolarchuk C., Scearce R., Foulger A., 2014 HIV-1 envelope gp41 antibodies can originate from terminal ileum B cells that share cross-reactivity with commensal bacteria. Cell Host Microbe. 16:215–226. 10.1016/j.chom.2014.07.003 25121750 PMC4294419

[bib42] Williams G.T., Jolly C.J., Köhler J., and Neuberger M.S. 2000 The contribution of somatic hypermutation to the diversity of serum immunoglobulin: dramatic increase with age. Immunity. 13:409–417. 10.1016/S1074-7613(00)00040-6 11021538

[bib43] Williams W.B., Liao H.X., Moody M.A., Kepler T.B., Alam S.M., Gao F., Wiehe K., Trama A.M., Jones K., Zhang R., 2015 Diversion of HIV-1 vaccine-induced immunity by gp41-microbiota cross-reactive antibodies. Science. 349:aab1253 10.1126/science.aab1253 26229114 PMC4562404

[bib44] Wilmore J.R., Gaudette B.T., Gomez Atria D., Hashemi T., Jones D.D., Gardner C.A., Cole S.D., Misic A.M., Beiting D.P., and Allman D. 2018 Commensal Microbes Induce Serum IgA Responses that Protect against Polymicrobial Sepsis. Cell Host Microbe. 23:302–311.e3. 10.1016/j.chom.2018.01.005 29478774 PMC6350773

[bib45] Xiao S., Brooks C.R., Zhu C., Wu C., Sweere J.M., Petecka S., Yeste A., Quintana F.J., Ichimura T., Sobel R.A., 2012 Defect in regulatory B-cell function and development of systemic autoimmunity in T-cell Ig mucin 1 (Tim-1) mucin domain-mutant mice. Proc. Natl. Acad. Sci. USA. 109:12105–12110. 10.1073/pnas.1120914109 22773818 PMC3409739

[bib46] Young G.R., Mavrommatis B., and Kassiotis G. 2014 Microarray analysis reveals global modulation of endogenous retroelement transcription by microbes. Retrovirology. 11:59 10.1186/1742-4690-11-59 25063042 PMC4222864

[bib47] Yu P., Lübben W., Slomka H., Gebler J., Konert M., Cai C., Neubrandt L., Prazeres da Costa O., Paul S., Dehnert S., 2012 Nucleic acid-sensing Toll-like receptors are essential for the control of endogenous retrovirus viremia and ERV-induced tumors. Immunity. 37:867–879. 10.1016/j.immuni.2012.07.018 23142781

[bib48] Zeng M.Y., Cisalpino D., Varadarajan S., Hellman J., Warren H.S., Cascalho M., Inohara N., and Núñez G. 2016 Gut Microbiota-Induced Immunoglobulin G Controls Systemic Infection by Symbiotic Bacteria and Pathogens. Immunity. 44:647–658. 10.1016/j.immuni.2016.02.006 26944199 PMC4794373

[bib49] Zuccarino-Catania G.V., Sadanand S., Weisel F.J., Tomayko M.M., Meng H., Kleinstein S.H., Good-Jacobson K.L., and Shlomchik M.J. 2014 CD80 and PD-L2 define functionally distinct memory B cell subsets that are independent of antibody isotype. Nat. Immunol. 15:631–637. 10.1038/ni.2914 24880458 PMC4105703

