# Supplementary material for: A splenic IgM memory subset with antibacterial specificities is sustained from persistent mucosal responses
Source: J Exp Med. 2018 Aug 6;215(8):2035–53. doi: 10.1084/jem.20180977 (PMC6080908; doi:10.1084/jem.20180977)
Supplement: Supplemental Materials (PDF) [file JEM_20180977_sm.pdf]

JEM

Supplemental material

Le Gallou et al., https://doi.org/10.1084/jem.20180977

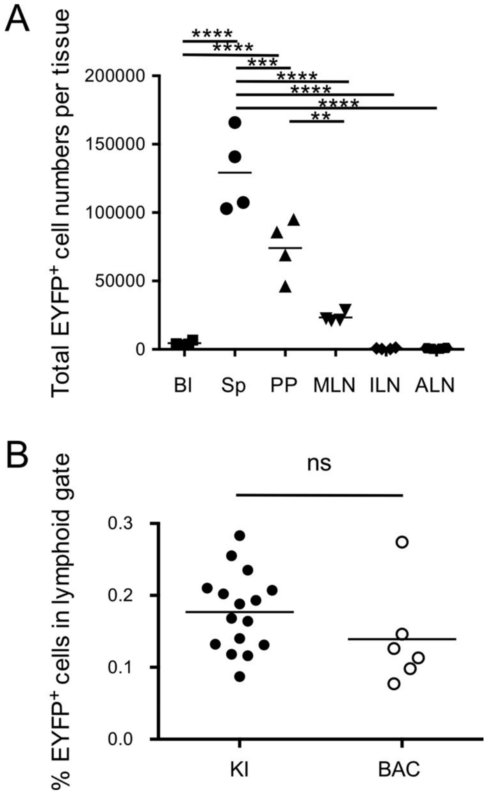

Figure S1. **EYFP⁺ labeling dominates in spleen compared with other tissues and is similarly observed in a BAC-AID-Cre-ERT2 mouse line (related to Fig. 1). (A)** The fraction of EYFP⁺ labeled cells was estimated in blood (Bl), spleen (Sp), PPs, MLNs, inguinal lymph nodes (ILNs), and axillary lymph nodes (ALNs) from the same mouse, 3 mo after tamoxifen feeding. **(B)** Percentage of EYFP⁺ labeling observed in spleen cells, 3–5 mo after tamoxifen feeding, in two different AID-Cre-ERT2 fate mapping mouse models. KI, model initially described by Dogan et al. (2009) used throughout this study, obtained by knock-in at the *Aicda* locus and showing *Aicda*-haploinsufficiency. BAC, BAC transgenic line, with the same AID-Cre-ERT2 modification inserted by homologous recombination in the 190-kb RP24-68I7 clone containing the *Aicda* locus, present as a one copy transgene. **, P < 0.01; ***, P < 0.001; ****, P < 0.0001, one-way ANOVA with Holm-Sidak correction (A); ns, not significant, Student's *t* test (B).

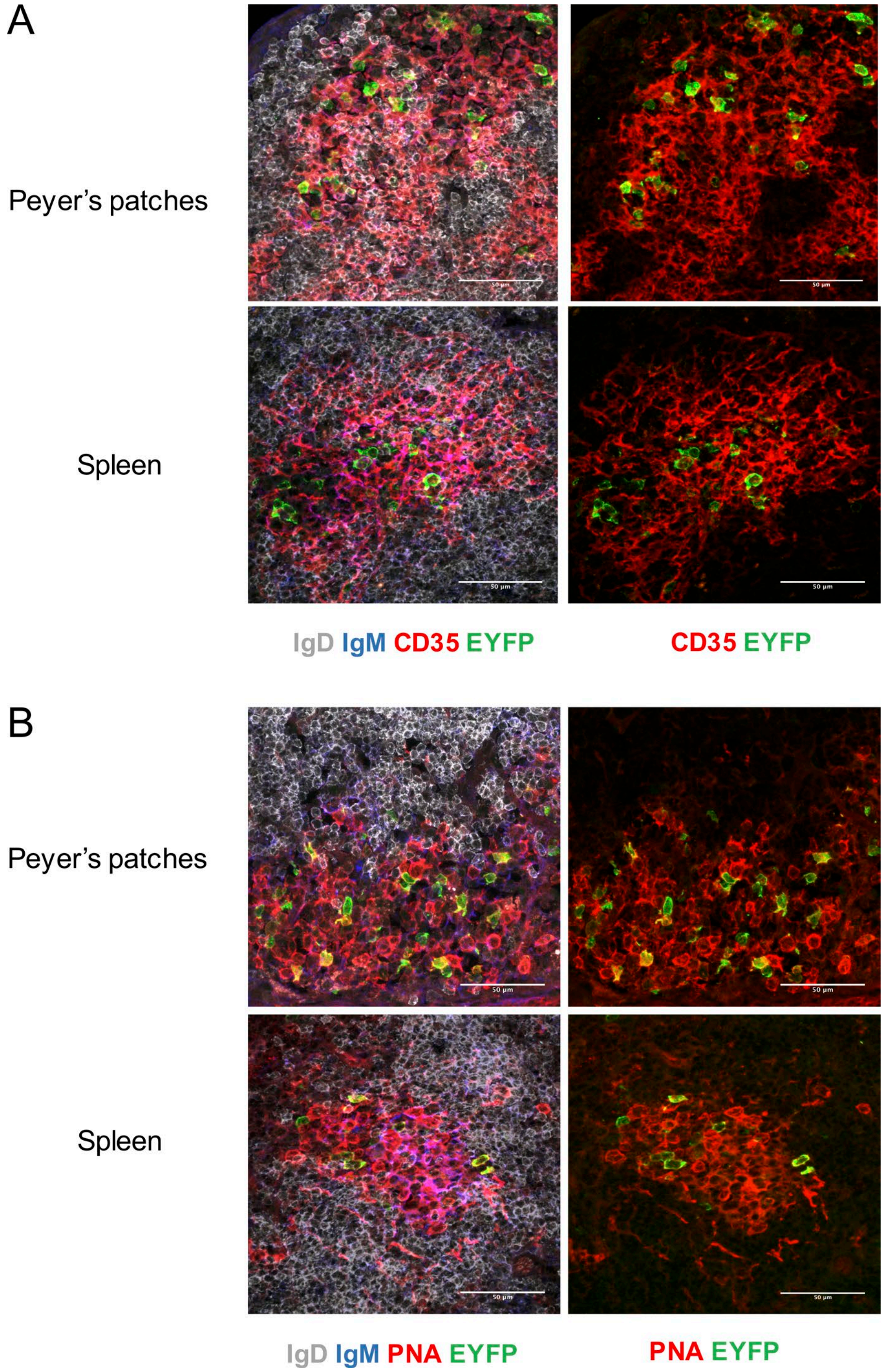

Figure S2. **EYFP-labeling takes place in GC B cells (related to Fig. 2). (A and B)** EYFP+ cells, 48 h after a single tamoxifen gavage, are localized mainly within CD35+, GC structures (A) and harbor a PNA+ phenotype (B) in both spleen and PPs. Bars, 50 µm. The four- or two-color staining is indicated below each image.

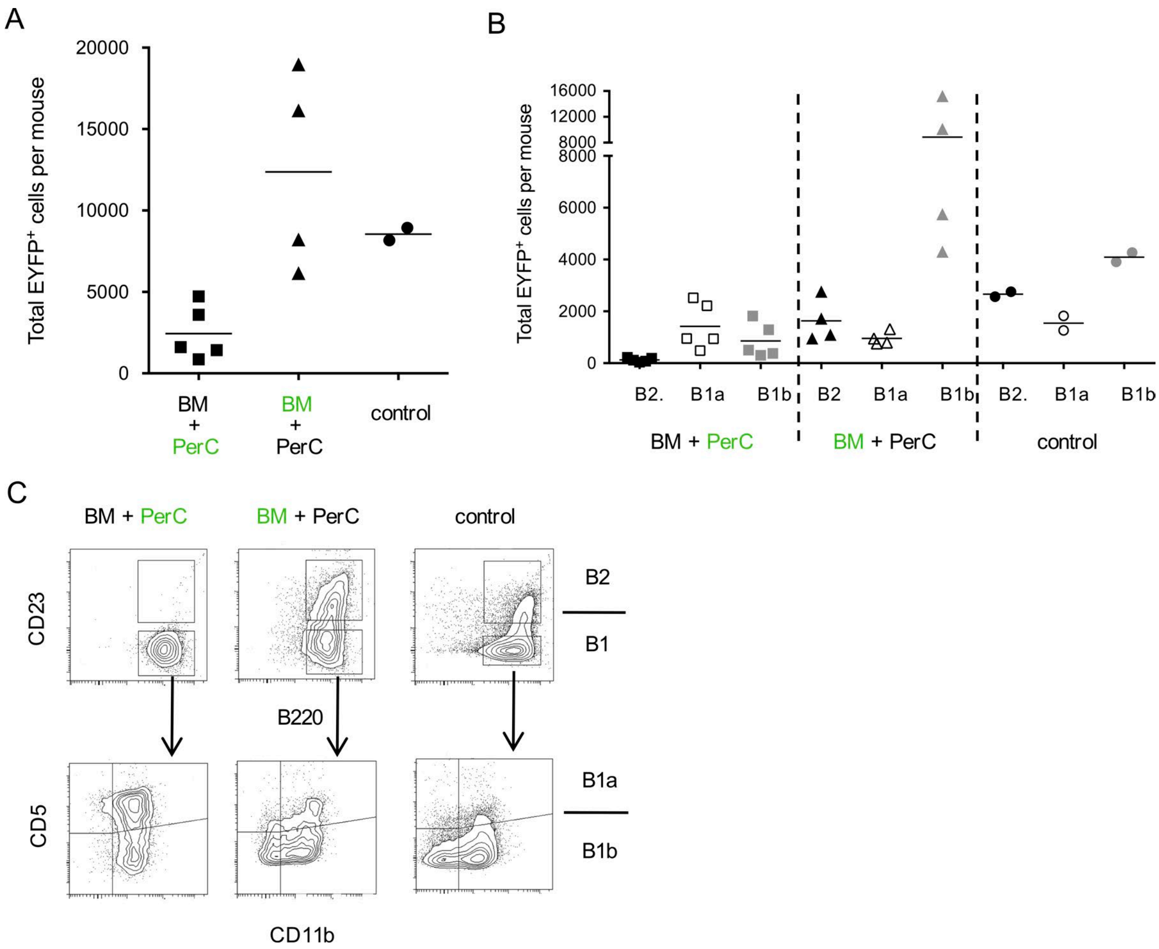

Figure S3. **A fraction of B1b cells expresses AID (related to Fig. 4). (A)** Analysis of the peritoneal cavity was performed in mice reconstituted according to scheme of Fig. 4 A. **(B)** Presence of the AID-reporter in BM donor cells allows the specific labeling of the B1b compartment. Mice fed with a similar tamoxifen regimen are shown as controls. **(C)** Representative FACS profiles of the B2, B1a, and B1b subsets in the peritoneal cavity, gated on EYFP+ cells. B1a and B1b cell counts featured in B are based on CD5+ and CD5− fractions represented in the bottom panels.

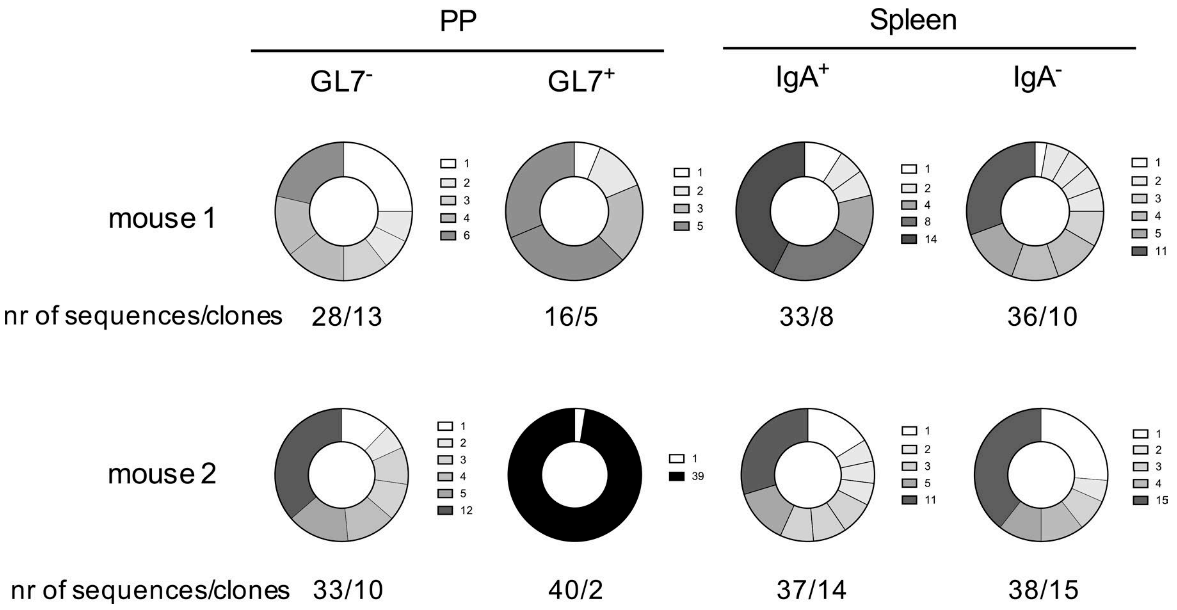

Figure S4. **Clonal distribution of PPs and splenic EYFP⁺ subsets recovered after anti-CD20 depletion (related to Fig. 7).** Sequences were obtained from two mice, 90 and 107 d after anti-CD20 depletion for four different subsets: B220⁺GL7⁺ and GL7⁻ from PPs and B220⁺IgA⁺ and IgA⁻ from spleen. Clonal distribution is depicted as in Fig. 6 A. Each pie section represents one clone, except for the white segment that represents all unique sequences. The total number of sequences and clones is indicated below each pie chart and the clone size is color-coded for each subset as indicated on the right side. Clonal relationships between subsets and tissues are represented in Fig. 7 E. nr, number.

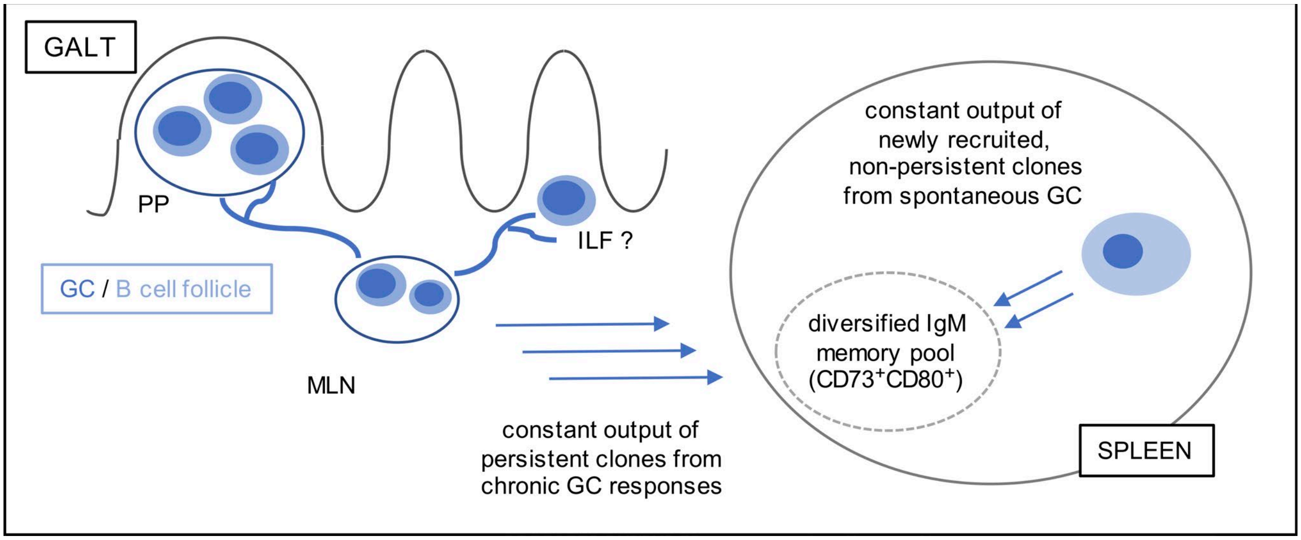

Figure S5. **The CD73⁺CD80⁺IgM⁺ subset, diversified by somatic mutation, is contributed by both spontaneous GCs from spleen and chronic GC reactions from PPs.** The CD73⁺CD80⁺IgM⁺ subset, which represents 4–6% of the spleen B cell pool of a 5–8-mo-old, adult mouse is contributed by constant export of persistent clones within mucosal immune responses (PPs, MLNs, and, possibly, isolated lymphoid follicles [ILF]), contributing a large fraction of the persisting EYFP⁺ pool, as well as by spontaneous GCs from spleen, in which clonal persistence is rarely observed.

Table S1. J_H4 intronic mutation frequency in $CD73^+CD80^+$ $EYFP^-$ IgM^+ splenic B cells (relates to Fig. 3)

Mouse age	Mutation frequency (total sequences)	Mutation frequency (mutated sequences)	% Mutated sequences
5 mo-1	0.25	0.62	40
5 mo-2	0.41	0.75	55
9 mo-1	0.32	0.59	55
9 mo-2	0.23	0.62	37
9 mo-3	0.49	0.68	62
9 mo-4	0.29	0.72	40

Table S2. **Hybridoma heavy chain characteristics (relates to Fig. 9)**

Hybridoma	VH gene	CDR3 aa sequence	Mutations	CDR3 length	N additions
**Conventional mice**					
C7	V7-3	ARYTDLLTDYAMDY	3	42	12
C11	V6-3	TDPGIDFDV	13	27	6
E9	V1-53	ARDCYGSDY	15	27	6
1B3	V3-6	ARRRDGYYGYFDV	10	39	6
1B5	V4-1	TRRGYGSHWYFDV	10	39	6
1C8	V7-1	ARNYYGSSY	3	27	0
1G2	V6-3	TGDYYGSRNY	2	30	4
1G8	V1-74	AMAPY	20	15	5
1G9	V6-3	TDPFKDY	9	21	10
1H2	V7-3	ARYLYDGYSPFAY	3	39	6
2D6`	V6-3	TDVTGTEFAS	11	30	6
		Mean	9.0	30.6	6.7
F7=E9; H10=C11; 1B8=1B5					
**Germ-free mice**					
A2	V6-3	TDPTVVPFAY	0	30	6
A6	V6-3	TDPGQAGTY	6	27	12
C2	V1-54	ARSSLGSLDY	3	30	11
C4	V1-50	ARRTWLRRFDY	0	33	7
D8	V1-31	ARSDEGFPY	3*	27	10
F9	V1-31	ARSDEGFPY	4*	27	10
H5	V1-53	TTYRPHYFDY	2	30	8
H6	V6-3	GTLVTTRYFDY	1	33	11
		Mean	2.4	29.6	9.4
C1=H5; E5=D8					

*D8 and F9 differ by five mutations and have only one in common.

Reference

Dogan, I., B. Bertocci, V. Vilmont, F. Delbos, J. Mégret, S. Storck, C.A. Reynaud, and J.C. Weill. 2009. Multiple layers of B cell memory with different effector functions. Nat. Immunol. 10:1292–1299. https://doi.org/10.1038/ni.1814

Table S3. **Antibody list (relates to Materials and methods)**

Antibody	Clone	Manufacturer	Catalog number
Anti-mouse B220	APC-eFluor780, Clone RA3-6B2	eBioscience	47-0452-82
Anti-mouse GL7	eFluor450, Clone GL7	eBioscience	48-5902-82
Anti-mouse GL7	PerCP-Cy5.5, Clone GL7	BioLegend	144610
Anti-mouse GL7	Biotin, Clone GL7	eBioscience	13-5902-85
Anti-mouse CD73	eFluor450, Clone eBioTY/118	eBioscience	48-0731-82
Anti-mouse CD80	APC, Clone 16-10a1	Sony Biotech	104714
Anti-mouse CD95	PE-Cy7, Clone Jo2	BD Biosciences	557653
Anti-mouse CD93	APC, Clone AA4.1	eBioscience	17-5892-83
Anti-mouse CD11b	APC-eFluor780, Clone M1/70	eBioscience	47-0112-82
Anti-mouse CD11c	AlexaFluor700, Clone N418	BioLegend	117320
Anti-mouse CD23	PerCP-Cy5.5, Clone B3B4	BioLegend	101617
Anti-mouse CD23	PE/Dazzle 594, Clone B3B4	BD Biosciences	563986
Anti-mouse CD21	PE/Dazzle 594, Clone 7G6	BD Biosciences	563959
Anti-mouse CD29	AlexaFluor 700, Clone HMb1-1	BioLegend	102218
Anti-mouse CD5	PE, Clone 53-7.3	BioLegend	553022
Anti-mouse CD43	BV510, Clone S7	BD Biosciences	563206
Anti-mouse CD49f	PE/Dazzle 594, Clone GoH3	BioLegend	313625
Anti-mouse CD62L	AlexaFluor 700, Clone MEL-14	BioLegend	104426
Anti-mouse CD9	BV711, Clone AA4.1	BD Biosciences	740696
Anti-mouse CXCR4	PE/Dazzle 594, Clone L276F12	BioLegend	146513
Anti-mouse BLIMP-1	PE, Clone 5E7	BioLegend	150005
Anti-mouse IgA	PE, Clone 11-44-2	Southern Biotech	1165-09
Anti-mouse IgA	Biotin, Clone 11-44-2	Southern Biotech	1165-08
Anti-mouse IgD	PerCP-Cy5.5, Clone 11.26c.2a	BD Bioscience	564273
Anti-mouse IgD	eFluor450, Clone 11.26c	eBioscience	48-5993-82
Anti-mouse IgG1	APC, Clone X56	BD Biosciences	550874
Anti-mouse IgG2a/c	PE, Clone RMG2a-62	BioLegend	407107
Anti-mouse IgG2b	PE, Clone RMG2b-1	BioLegend	406708
Anti-mouse IgG3	PE, Clone SB76b	Southern Biotech	1191-09
Anti-mouse IgM	BV605, Clone RMM-1	BioLegend	406523
Anti-mouse IgM	Cy5, goat polyclonal	Southern Biotech	1020-15
Anti-mouse PD-L1	PE, clone MIH5	BD Biosciences	12-5982-81
Anti-mouse PD-L2	BV510, clone TY25	BD Bioscience	740194
Anti-mouse Ki-67	Alexa Fluor 555, Clone B56	BD Bioscience	558617
Anti-mouse a4b7	BV711, Clone DATK32	BD Bioscience	740701
Anti-mouse Tim-1	Biotin, Clone RMT1-4	BioLegend	119503
Anti-mouse CD267	PE, Clone ebio8F10-3	eBioscience	12-5942-81
Anti-GFP	uncoupled, chicken polyclonal	Abcam	ab13970
Anti-chicken Ig	AlexaFluor 488, goat polyclonal	Invitrogen	A11039
Anti-mouse CD35	Biotinylated (rat), clone 8C12	BD Biosciences	553816
Anti-mouse CD20	Clone 18B12	Biogen Idec	
**Other staining reagents**			
Peanut Agglutinin (PNA)	Biotin	Vector Laboratories	B-1075
Streptavidin	AlexaFluor 555	Invitrogen	S21381
Streptavidin	PE-Cy7	Sony Biotechnology	2626030
7-AAD viability solution		Biolegend	420403
